# The Dose-Related Efficacy of Acupuncture on Endometrial Receptivity in Infertile Women: A Systematic Review and Meta-Analysis

**DOI:** 10.3389/fpubh.2022.858587

**Published:** 2022-04-28

**Authors:** Xiaoyan Zheng, Siyi Yu, Liying Liu, Han Yang, Fangge Wang, Hongmei Yang, Xingyu Lv, Jie Yang

**Affiliations:** ^1^Acupuncture and Tuina School, Chengdu University of Traditional Chinese Medicine, Chengdu, China; ^2^Clinical Research Center for Acupuncture and Moxibustion in Sichuan Province, Chengdu Xi'nan Gynecological Hospital, Chengdu, China

**Keywords:** dose-related, acupuncture, endometrial receptivity, meta-analysis, heterogeneity analysis

## Abstract

**Background:**

Progress has been achieved by using acupuncture widely for poor endometrial receptivity (PER). However, different acupuncture dosages may lead to controversy over efficacy.

**Objective:**

To evaluate the evidence-based conclusions of dose-related acupuncture on infertile women with PER.

**Method:**

References were retrieved from nine databases from inception to 26 February 2022. This meta-analysis included randomized controlled trials (RCTs) that investigated the dose-related efficacy of acupuncture for PER with outcomes of endometrium receptivity (ER) parameters by transvaginal sonography (TVS) and the subsequent pregnancy outcomes in three acupuncture-dose groups: the high-dosage group (three menstrual cycles), the moderate-dosage group (one menstrual cycle), and the low-dosage group (two or four days). Since there remained sufficient heterogeneity among the three subsets, we prespecified seven subgroup variables (four clinical and three methodological) to investigate the heterogeneities.

**Results:**

A total of 14 RCTs (1,564 women) of moderate or low overall quality were included. The results were different when the dosage of acupuncture was restricted. For the moderate or high-dosage group, CPR and part of ER parameters were improved in the acupuncture group (i.e., CPR: OR = 2.00, 95% CI [1.24, 3.22], *p* = 0.004, *I*^2^ = 0% in one menstrual cycle; OR = 2.49, 95%CI [1.67, 3.72], *p* < 0.05, *I*^2^ = 0% in three menstrual cycles). However, for the low-dosage group, no statistical difference was observed in CPR (OR = 0.07, 95% CI [−0.10, 0.23], *p* = 0.44, *I*^2^ = 82%) and a part of the ER parameters. In subgroup analysis, four subgroup variables (the routine treatment, risk of performance bias, duration of acupuncture treatment, and the age of participants) could explain some of the heterogeneities across all trials.

**Conclusion:**

The finding indicated that the trend of relatively more acupuncture dosage showed better effects for poor endometrial receptivity among PER women. It remains a potential heterogeneity in our studies. Further high-quality trials with a homogeneity trial design need to be conducted.

## Introduction

The incidence of infertility has begun to increase annually (1, 2), and it has gradually become the third most common disease worldwide (3). Potential embryonic development, optimal endometrial receptivity (ER), and synchronization of the embryo and endometrium play critical roles in a successful pregnancy (4). ER refers to the endometrium's ability to allow the blastocyst to attach and grow. Impaired ER will reduce the synchronization and lead to infertility. Approximately 2/3 of embryo implantation (ET) failure is closely related to poor endometrial receptivity [PER; (5, 6)]. However, little progress has been achieved for PER over three decades after the introduction of *in vitro* fertilization (IVF), which has achieved great improvement in embryo quality (4).

Acupuncture has been widely used to treat infertility for a long period. Some potential mechanisms have been postulated to explain the role of acupuncture in the IVF procedure. Firstly, for the stimulation of ovary induction, electro-acupuncture can alter several different neuroendocrinological factors, such as β-endorphin, which can mediate the hypothalamus-pituitary-gonadal (HPG) and-adrenal axes (HPA) and regulate the menstrual cycle, ovulation, and fertility (7). Secondly, electro-acupuncture can circulate the blood flow of the uterus, reduce the resistance of uterine arteries (8), and increase ovarian blood flow through the ovarian sympathetic nerves (9). Thirdly, acupuncture may have the efficacy of meditating the immune response for the achievement and maintenance of a successful pregnancy (10). Besides, acupuncture can reduce the anxiety level (11) and regulate serum cortisol (CORT) and PRL (12), which simulate estradiol (E2) and progestin (P) independently. However, the dosage of the acupuncture intervention varies from 2 (13) to 18 sessions (14) (lasting for three menstrual cycles), and the efficacy of acupuncture remains a contention worldwide. In 2002, Paulus et al. (13) conducted the first randomized controlled trial (RCT) which evaluated the efficacy of acupuncture for women undergoing *in vitro* fertilization and embryo transfer (IVF-ET) with two sessions of acupuncture (25 min before ET and after ET) and found out that acupuncture could significantly improve clinical pregnancy rate (CPR), which was 42.5% in the acupuncture group compared to 26.3% in the control group. However, many trials (15–18) which attempted to repeat this outcome with the same intervention (2 sessions of acupuncture) have not been successful. Moreover, Ajeena et al. (19) found out that six sessions of transcutaneous electrical nerve stimulation (TENS), which is a kind of electro-acupoint stimulation, are beneficial to increase endometrial thickness in healthy women at their childbearing age. For the duration of three menstrual cycles of acupuncture, Zhuang et al. (20) found that CPR and EMT were improved significantly, while Shuai et al. (14) did not find out the same outcome in EMT. Furthermore, Belinda (21) confirmed that infertility is a complex medical issue, often associated with either significant previous gynecological issues/pathology and/or with advanced-age patients (>30 y or <40 y). Infertile women who receive longer-term treatment may benefit from the effects of regular acupuncture on their other health issues. But it did not mention the optimal dosages of acupuncture.

Therefore, the purpose of this systematic review is to evaluate the efficacy of acupuncture in improving the ER for infertile women with PER and to identify the optimal acupuncture plan (in terms of duration of acupuncture intervention, and optimal intervention measurement). The finding can be used to obtain more vigorous evidence-based clinical practice.

## Method

### Protocol and Registration

The systematic review protocol has been registered on the prospective international register of systematic review (PROSPERO: registration number is CRD42020206790) (https://www.crd.york.ac.uk/PROSPERO/).

All contents and report details were strictly referred to as Preferred Reporting Items for Systematic Reviews and Meta-analyses (PRISMA) (22), as shown in Supplementary Material S1.

### Literature Search Strategy

References were retrieved from nine databases: four English databases (i.e., PubMed, Embase, Cochrane Library, and Web of Science), and five Chinese databases, i.e., SinoMed (formerly Chinese Biomedical Database), Chinese National Knowledge Infrastructure (CNKI), Wanfang Data, China Biomedical Literature Database, and China Science Journal Database (VIP database) from inception to 25 February 2022. The search strategy was based on the guidance of the Cochrane handbook. The language was limited to English and Chinese. The search strategies are shown in Supplementary Material S2.

In addition, to reduce the publication bias, we searched for lists of relevant references. Clinical trial registries, i.e., Menstrual Disorders and Subfertility Group (MDSG) Specialized Register, Cochrane Central Register of Controlled Trials (CENTRAL), World Health Organization International Clinical Trials Registry Platform, Chinese clinical registry, and Clinical Trials. Gov, and we manually searched key journals and meetings such as the European Society for Human Reproduction and Embryology (ESHRE) and American Society of Reproduction Medicine (ARSM) for relevant articles, including relevant journals and conferences abstracts, by connecting with the coordinator.

### Inclusion and Exclusion Criteria

#### Study Participants

In our study, the starting points are those patients who suffered from PER where the uterine factor is routinely evaluated by transvaginal sonography (TVS) without anatomic abnormality. Repeated implantation failure (RIF) (23) and polycystic ovarian syndrome (PCOS) (24) were included considering impaired ER can be found in the categories of patients.

#### Study Intervention and Comparison

We collected randomized controlled trials (RCTs) that compared verum acupuncture with placebo acupuncture, or no adjuvant treatment in the review. In a broad sense, verum acupuncture included auricular acupuncture, electro-acupuncture, manual acupuncture, and transcutaneous electrical acupoint stimulation (TEAS) with or without moxibustion. Placebo acupuncture included placebo acupuncture devices, shallow acupuncture, non-acupoints or non-therapeutic acupoints, and mock electrical stimulation. No adjuvant treatment only included western routine treatment.

#### Study Outcomes Measures

The primary outcome was the clinical pregnancy: the presence of at least one intrauterine gestational sac or fetal heartbeat confirmed by ultrasound 4–6 weeks after embryo transfer or ovulation, and at least one of the following outcomes was extracted as the secondary outcomes by TVs:

- EMT: It refers to the distance between the endometrium's anterior and posterior walls, including the uterine cavity gap. EMT is one of the most frequently employed indirect predictors of ER. The pregnancy outcome was significantly higher in women with 7 < EMT ≤ 14 mm (25). However, EMT is a conflicting indicator in predicting the pregnancy outcome alone (26).
- Endometrium pattern (EMP), according to Gonen's criterion (27): Type A, trilinear or multilayered endometrium, strong echo in the outer and moderate parts, hypoechoic or dark areas in the inner layer, and unmistakable linear echo in the uterine cavity; Type B, weak trilinear, isolated echo in the moderate, inconspicuous echo in the moderate uterine cavity; and Type C, strong echo, no intrauterine midline echo. Type A with “triple-line” appears important in improving the pregnancy rate (25).
- Blood flow indicators include the resistance index (RI), pulse index (PI), endometrial vascular index (VI), flow index (FI) of the uterine artery, and endometrial blood flow: peak systolic velocity/ end-diastolic blood velocity (S/D) (28). Reducing uterine vascular RI and improving uterine blood flow can improve the implantation rate (29).
- Live birth: The live birth is newborns (>28 weeks of gestation) who were delivered with signs of life. Live birth rate (LBR is a ratio of live birth and clinical pregnancy).

We excluded RCTs that extracted EMT as the only outcome after acupuncture treatment because the utilization of EMT as a tool to decide on IVF cycle cancellation is not justified based on the current meta-analysis (30). The Chinese herb utilization was excluded to evaluate the efficacy of acupuncture precisely.

### Selection of Studies and Data Extraction

Two reviewers (XYZ and HMY) independently selected the studies, extracted data, and downloaded the citations into Note Express software Version 2.6.1 (Aegean Sea software company, Beijing, China) for data management and eliminated the duplicate research by software. Any discrepancies were resolved through further discussion with the third reviewer (FGW).

For trials to be eligible, intervention dosage (duration and frequency) for acupuncture treatment is extracted in detail. We categorized the acupuncture dosages into three groups: low, moderate, and high. Moderate-dosage group was defined as “one menstrual cycle” of acupuncture duration [including the acupuncture conducted during the controlled ovarian hyperstimulation (COH) procedure in IVF or during frozen-thawed embryo transfer (FET)]. For the duration of acupuncture that was less than “one menstrual cycle,” we extracted studies in the “low-dosage group,” while for acupuncture duration that was more than “one menstrual cycle,” we extracted the studies in the “high-dosage group.”

### Assessment of Included Studies

#### Assessment of Bias

The risks of bias of RCTs were assessed using the Cochrane Collaboration's tool (31). The criteria consist of seven items: i) selection bias (random sequence generation and allocation concealment); ii) performance bias (blinding of participants and personnel); iii) detection bias (blinding of outcome assessment); iv) attrition bias (incomplete outcome data); v) reporting bias (selective reporting), and vi) other bias. Each study was evaluated as high, moderate, low, or very low for each evidence.

#### The Quality of Evidence

We used the Grades of Recommendation, Assessment, Development, and Evaluation [GRADE; (32)] score to assess the quality of each piece of evidence. The quality was classified into high, moderate, or very low, and five reasons to possibly rare down the quality of each evidence are as follows: i) limitation in study design or execution (risk of bias); ii) inconsistency of results, inconsistent results; iii) evidence of indirectness; iv) imprecision; v) publication bias. Each study was evaluated as high, low, or unclear risk of bias for each item.

### Data Synthesis and Analysis

We used the RevMan5.4.1 provided by the Cochrane Collaboration to analyze data. For dichotomous data (CPR, LBR, and EMP), we expressed the results for each study as the odds ratio (OR) with a 95% confidence interval (CIs). For continuous data (EMT, RI, PI, and S/D), we expressed the results as the difference or standardized mean difference (SMD) with 95% CI. If data could be synthesized, we used descriptive analysis to solve this problem. For pooled data, we used *I*-square (*I*^2^) statistics, which indicates the proportion of variability across trials not explained by sampling variation alone, and the Cochran Q-test for heterogeneity assessing (31). A fixed-effects model was performed for low heterogeneity (*I*^2^ < 50% statistics or the Cochran Q-test, *p* > 0.05), otherwise, random-effects were performed. We used network construction between acupoints-associated and duration-associated to show their relationship visually.

To find out the dose-related efficacy of acupuncture, we divided the extracted data into three groups based on the different dosages of acupuncture intervention separately (2 or 4 days only, one menstrual cycle, or three menstrual cycles). We assessed the likelihood of publication bias by constructing funnel plots and the Eggers' test.

### Subgroup Analyses

For pooled data, characteristics varied from different studies. We used subgroup analysis instead of meta-regression to find the source of heterogeneity, mainly considering the insufficient number of included studies. Subgroup analysis was based on four clinical characteristics: i) acupuncture duration; ii) intervention of different routine treatment (IVF-ET, FET, or clomiphene citrate (CC)/letrozole (LE) for ovulation induction); iii)acupuncture used alone or with other intervention; iv) mean age of participants (≥35 years or not) and three methodological characteristics: i) risk of blinding of participants and personnel (low risk or high risk); ii) risk of random sequence generation (low risk or high risk); and iii) risk of allocation concealment (low risk or high risk).

For each subgroup analysis, we performed a single covariate weighted random effects with 95% confidence interval in Stata version 16 (*StataCorp)* to investigate whether differences in effects of adjuvant acupuncture between the covariate's two subgroups were statistically significant. For each single covariate subgroup analysis, we calculated the interaction *p*-value of the test and the percentage of the heterogeneity explained by the covariate (*I*^2^).

## Results

### Selection of Studies

**Figure 1:**
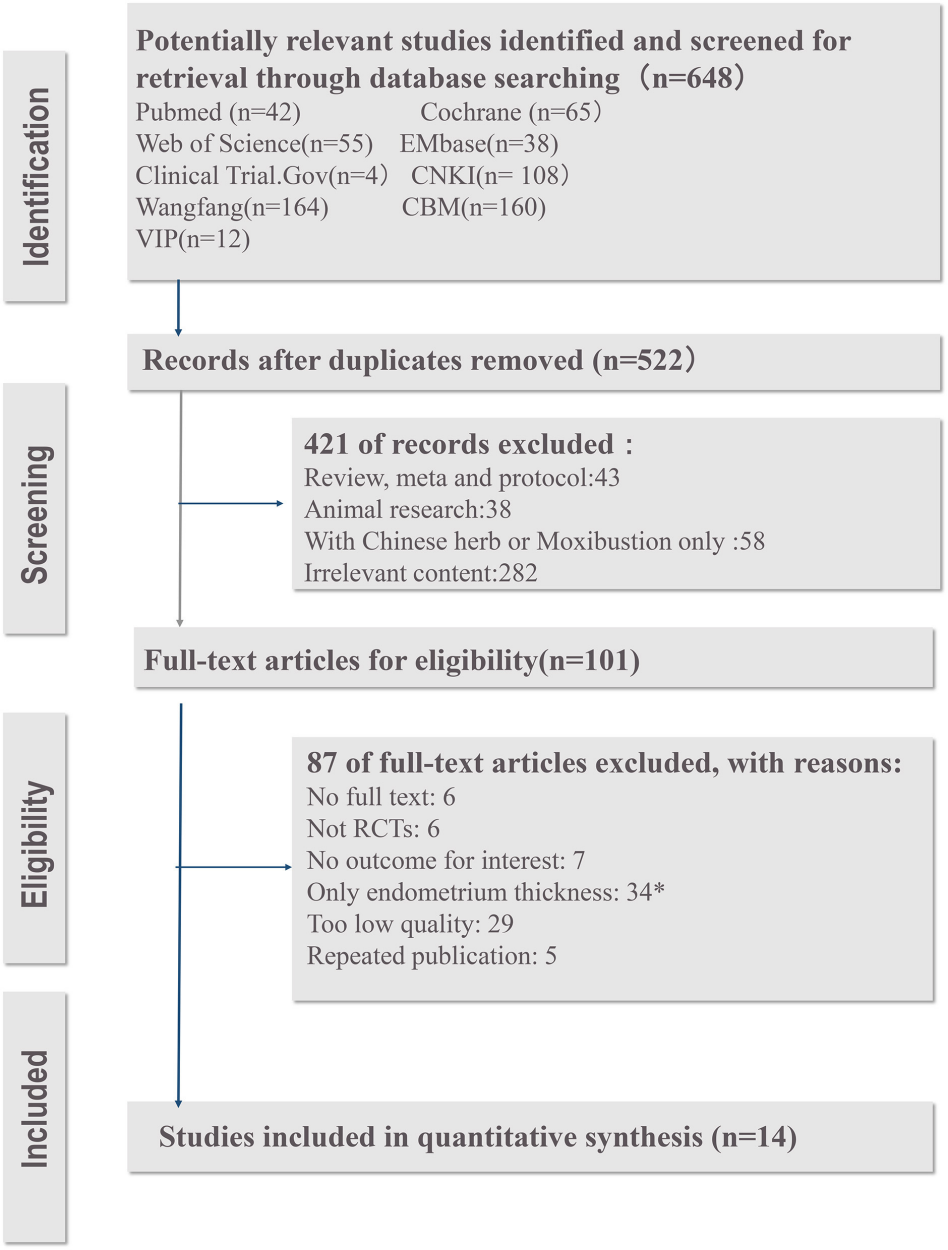
The PRISMA flow diagram of study screening process. ^*^The studies were excluded because that the utilization of EMT as a tool to decide on IVF cycle cancelation is not justified based on the current meta-analysis.

Figure 1 shows the details of the study selection process.

The PRISMA flow diagram (Figure 1) shows the details of the study selection proces. Of the 101 identified trials, 76 studies were excluded after screening full-text (6 studies were not available for full-text. Six studies were not RCTs, seven studies had no outcomes for interests, 34 studies had the only outcome of EMT, which cannot represent ER in our opinion, and 34 other studies were also excluded because of low quality or repeated publication). Thus, 14 RCTs with a total of 1,564 participants met the inclusion criteria. Twelve (14, 16, 20, 33–41) studies were conducted in China [ten studies (14, 20, 33, 34, 36–41) were from the mainland, China and two were (16, 35) from Taiwan, China], and two (13, 42) were from Germany.

### Trial Characteristics

Table 1 shows the characteristics of RCTs included in the review. The differences in trial eligibility criteria were that six trials (14, 20, 34, 36, 39, 41) included women with RIF and one trial (37) included women with PCOS. The duration of the acupuncture sessions differed among trials (Table 1). Three studies (13, 16, 42) conducted two sessions around ET and one study (35) conducted 4 sessions during COH. Some women received acupuncture for almost one menstrual cycle [acupuncture was performed for 1 month before FET in one study (38) and during FET in three studies (36, 39, 40)]. Meanwhile, the other six trials (14, 20, 33, 34, 37, 41) lasted for three menstrual cycles before embryo transfer. Six studies (13, 16, 33, 35, 38, 42) conducted IVF-ET of routine treatment, seven studies (14, 20, 34, 36, 39–41) were performed in FET, and one study (37) administered letrozole (LE) and human chorionic gonadotropin (HCG) for the induction of ovulation in routine treatment.

**Table 1:** Characteristics of included trials.

**References**	*N* ^*^	**Study population**	**Mean age**	**Acupuncture group**	**Control group^†^**	**Starting time of intervention^§^ And the duration of intervention**	**Outcomes**	**Routine treatment**
Chen and Hau (33)	T:57 C:57	i)age: 24–35y ii)IVF-ET iii)regular menstrual cycles	T:31 ± 3 C:31 ± 3	**Acupuncture& Moxibustion (*****n*** **=** **57)** i)Acupuncture: [Needles stimulated manually by rotating, lifting, and thrusting the handle of the needle to maintain de qi sensation, both during initial insertion and after 10 min] ii)Moxibustion: [Light the moxibustion strip to navel ^¶^ until the patient feel warm for 30 min]	**No adjuvant treatment (*****n*** **=** **57)**	**Ac time:** during COH **Frequency**: every day **Duration:**3 menstrual cycles	EMT, EMP, S/D, PI, RI	IVF-ET
Paulus et al. (13)	T:80 C:80	i)IVF-ET	T: 32.1 ± 3.9 C: 32.8 ± 4.1	**Acupuncture& auricular acupuncture (*****n*** **=** **80)** i) Acupuncture: [soreness, numbness, or distention around the point = Deqi sensation) occurred during the initial insertion.]	**No adjuvant treatment (*****n*** **=** **80)**	**Ac time:** around ET **Frequency:** 25 min before and after embryo transfer **Duration:**2 days	EMT, PI, CPR	IVF-ET
Ho et al. (35)	T:30 T:26	i)IVF-ET	T: 35.5 ± 4.5 C: 34.0 ± 5.2	**Electro-acupuncture (*****n*** **=** **30)** [The needles were twirled by hand to evoke a needle reaction; this often resulted in soreness, numbness, and distension around the point, The needles were then attached to an electrical stimulator at a low frequency of 10 Hz for 30 min]	**No adjuvant treatment (*****n*** **=** **14)**^‡^	**Ac time:** during COH (from day 2 of the study to the day before oocyte retrieval) **Frequency:** Twice a week for 2 weeks **Duration:**4 days	PI, CPR	IVF-ET
Dieterle et al. (42)	T:116 C:109	i)infertility	T: 35.1 ± 3.8 C:34.7 ± 4.0	**Acupuncture & auricular acupuncture (*****n*** **=** **116)** i)Acupuncture: [soreness, numbness, or distention around the point = Deqi sensation) occurred during the initial insertion.] ii) auricular acupuncture: [a special Chinese medical drug (the seed of Caryophyllaceae) was placed on the patient's ears. The seeds remained in place for 2 days and were pressed twice daily for 10 min. 3 days after ET, all patients received a second acupuncture treatment.]	**Placebo acupuncture & placebo auricular acupuncture (*****n*** **=** **109)** i)Placebo acupuncture: [the same needle reaction was utilized as the acupuncture group with acupoints designed not to influence fertility] ii) placebo auricular acupuncture: [the same needle reaction was utilized as the acupuncture group with acupoints designed not to influence fertility]	**Ac time:** after ET **Frequency:** immediately after ET and again 3 days later **Duration:** 2 days	EMT, CPR	IVF-ET
Zhong ^**^ et al. (43)	T:51 C:52	i)age <35y ii)IVF-ET iii)normal BMI	T:31.37 ± 2.91 C:33.19 ± 2.57	**TEAS (*****n*** **=** **50)** ^**g**^ [TEAS (*HANS, Beijing, China*) stimulated via waves at 2 Hz frequency, 8–25 mA, which was the level of maximal tolerance without discomfort.]	**Mock TEAS (*****n*** **=** **50)**^††^ [Ineffective electrical stimulation]	**Ac time:** during FET (from day 2 or 3 of each cycle to 1 day before ET) **Frequency:** every day **Duration**:1 menstrual cycle	EMT, EMP, RI, PI, EMB, S/D	FET
Wang et al. (41)	T:30 C:30	i)infertility ii)RIF	T:35.0 ± 3.71 C:34.7 ± 3.18	**Acupuncture& Moxibustion (*****n*** **=** **30)** i) Acupuncture: [Needles manipulated to obtain de qi for 30min] ii)Moxibustion: [Burned moxa stuck at the top of needles]	**Sham acupuncture& Placebo moxibustion (*****n*** **=** **30)** i)Sham acupuncture: [Acupoint pressing stimulated epidermal irritation slightly without de qi.] ii)Placebo moxibustion: [Electromagnetic waves bake electric lamp produced the sensation of heat only.]	**Ac time:** three courses before FET (one course: 10 days before the period starting treatment to the day before the next menstruation) **Frequency:** not mentioned **Duration**:3 menstrual cycles	EMP, RI, PI, CPR	FET
Shuai et al. (14)	T:34 C:34	i)age: 25–40 y ii)regular menstrual cycles iii)RIF	T: 29.47 ± 3.24 C: 29.65 ± 2.60	**TEAS (*****n*** **=** **34):** [TEAS (*LH202H HANS, Huawei Co Ltd, Beijing, China*) using dispersed-dense waves at 2 Hz frequency. The intensity was set to approximately 10–20 mA]	**Mock TEAS (*****n*** **=** **34)** [TEAS electrodes applied to the same sites and received intermittent 2 Hz (10 s on and 20 s off) TEAS at an intensity of 5 mA.] ^h^	**Ac time:** three menstrual cycles before the scheduled FET **Frequency:** six times per cycle **Duration:** 3 cycles (18 treatment sessions in all).	EMT, EMP, CPR, LBR	FET
Zhao et al. (39)	T:38 C:34	i)age <38y ii)RIF	T:32.57 ± 4.25 C:33.71 ± 4.22	**Electro-acupuncture (*****n*** **=** **38):** [Needles manipulated to obtain de qi and connected electric-wire, the density wave (2/80 Hz) was set to the maximum comfort intensity that the patient could tolerate]	**Shallow acupuncture (*****n*** **=** **34):** [Shallow needles (depth <5 mm) placed on the non-acupoints, and not manipulated to achieve “de qi” sensation. Electrode wires connected with the shallow needles without electrical current]	**Ac time:** during FET (from day 2 or 3 of each cycle to 1 day before ET) **Frequency:** every other day **Duration:**1 menstrual cycle	EMT, EMV, FI, RI, VFI, CPR	FET
Zhuang (20)	T:36 C:36	i)age:24–45 ii)RIF	T: 34.2 ± 5. 31 C: 34.26 ± 5.30	**Acupuncture & Moxibustion & Cupping (*****n*** **=** **34)** ^**||**^ i)Acupuncture: [Needles manipulated to obtain de qi for 30min] ii)Moxibustion: [Burned moxa stuck at the top of needles] iii)Flash Cupping: [Flash cupping stimulated at abdominal acupoints until flushing skin during the follicular phase and luteal phase.]	**No adjuvant treatment (*****n*** **=** **35)** ^**||**^	**Ac time:** three menstrual cycles before the scheduled FET **Frequency: three** times a week **Duration:**3 menstrual cycles	EMT, EMB, RI, PI, CPR, LBR	FET
Ma and Zhang (36)	T:35 C:35	i)age:25–40y ii)RIF iii)BMI: 18.5~23.9 kg /m^2^ iv) more than two high-grade embryos remained	T:30.04 ± 2.98 C: 30.55 ± 3.71	**Acupuncture (*****n*** **=** **35)** [Needles stimulated manually by rotating, lifting, and thrusting to obtain de qi until the maximum comfort intensity that the patient could tolerate, different acupoints selected before and after embryo transfer.]	**No adjuvant treatment (*****n*** **=** **35)**	**Ac time:** during FET and immediately after ET **Frequency**: every other day **Duration:**1 menstrual cycle	EMT, RI, PI, CPR	FET
Chen and Hau (33)	T:25 C:31	i) age:22–40y ii)RIF iii)EMP:C type	T:35.64 ± 3.73 C:36.0 ± 2.98	**Acupuncture & Moxibustion (*****n*** **=** **25)** i)Acupuncture: [Needles manipulated to obtain de qi for 30 min] ii)Moxibustion: [Burned moxa stuck at the top of needles]	**No adjuvant treatment (*****n*** **=** **31)**	**Ac time:** three menstrual cycles **Frequency**: every day **Duration:**3 menstrual cycles	EMT, EMP, RI, PI, CPR	FET
So (16)	T:185 C:185	i) normal uterine cavity	A: 35.64 ± 3.73 C: 36.0 ± 2.98	**Acupuncture (*****n*** **=** **185)** [Needle reaction (soreness, numbness, or distension around the puncture sites or sometimes propagate along the corresponding meridians which termed the DeQi sensation) was elicited during the initial insertion]	**Placebo acupuncture (*****n*** **=** **185)** [The Streitberger's placebo needles were used, which was blunt. When it was pushed forward against, the skin, the needle slid into the handle and the whole needle appeared shortened.]	**Ac time:** around ET **Frequency:** 25 min before and after embryo transfer **Duration**:2 days	VI, FI, VFI, SEVI, SEVFI, SEFI, CPR, LBR	IVF-ET
Lin et al. (37)	T:35 C:35	i) age:20~40y ii)PCOS (based on Rotterdam criteria, 2003)	T: 27.74 ± 3.07 C: 26.71 ± 2.98	**Electro-acupuncture & Moxibustion(n=32)** i)Electro-acupuncture: [Filiform needles were used at different acupoints with continuous wave, 2 Hz in frequency and tolerable current intensity based on different phase of menstrual cycle.] ii)Moxibustion: [ginger-isolated moxibustion: the moxa cone was placed on the ginger and ignited with red skin in the local area, once every two days, 30 min each time]	**No adjuvant treatment (n=35)**	**Ac time:** during COH **Frequency:** every two days **Duration**:3 menstrual cycles.	EMT, RI, PI, S/D, CPR	letrozole and HCG
Zhong et al. (38)	T:32 C:32	i)infertility ii)age:35–42y iii)underwent IVF-ET	T: 36.52 ± 2.11 C: 36.19 ± 1.95	**Electro-acupuncture (*****n*** **=** **30)**^‡‡^ [Needles manipulated to obtain de qi and connected electrode wires, dilatational wave, maximum comfort intensity that the patient could tolerate for 30min, every other day.	**No adjuvant treatment (*****n*** **=** **31)**^‡‡^	**Ac time:** 1 menstrual cycle before the scheduled IVF-ET **Frequency: three** times a week **Duration:1** cycle	EMT, EMP, PI, RI, S/D, CPR	IVF-ET

* *Number randomized*;

† *For the control group trials, the procedure was given the same as the acupuncture group*;

§ *Based on the start time of the first acupuncture session: in menstruation or after menstruation*;

¶ *Navel is an acupoint of Ren meridian based on Traditional Chinese Medicine (TCM)*;

‡ *12 patients in the control group were excluded from the study: four of them failed ovarian stimulation and the other eight participants were declined to participate further after randomization*;

** *Three groups were set in the trial, TESA vs. Mock TESA vs. no adjuvant control group, we collected the first group and the second group in our study*;

†† *One patient in acupuncture group quitted and two patients in control group fell out due to incomplete data; || Two patients in the acupuncture group interrupted treatment because of out of work and one patient in the control group embryo transferred advanced*;

‡‡ *In acupuncture group, one patient dropped out and one was excluded due to insufficient data. In the control group, one patient dopped out*.

T, the treatment group; C, the control group; COH, control ovarian hyperstimulation; Ac, acupuncture; EMT, endometrial thickness; EMP, endometrial pattern; S/D, peak systolic velocity/ end-diastolic blood velocity; EMB, endometrial blood; RI, resistive index; PI, pulse index; IVF-ET, in vitro fertilization and embryo transfer; ET, embryo transfer; CPR, clinical pregnancy rate; TEAS, transcutaneous electrical acupoint stimulation; RIF, repeated implantation failures; HCG, human chorionic gonadotropin; PCOS, polycystic ovarian syndrome,^‡^§, ¶, ||, ^**^,^††^,^‡‡^.

For the frequency of the collected studies, one study (41) did not mention the frequency of acupuncture, three studies (33, 34, 40) conducted acupuncture every day, three studies (36, 37, 39) were conducted every other day, two studies (20, 38) were conducted three times a week, while one study (14) conducted for six times per cycle. However, all of the collected studies did not mention the total dosage of acupuncture treatments, except that three studies (13, 16, 42) were conducted only for 2 days (25 min before and after ET), and one study (35) was conducted for 4 days in total.

**Figure 2:**
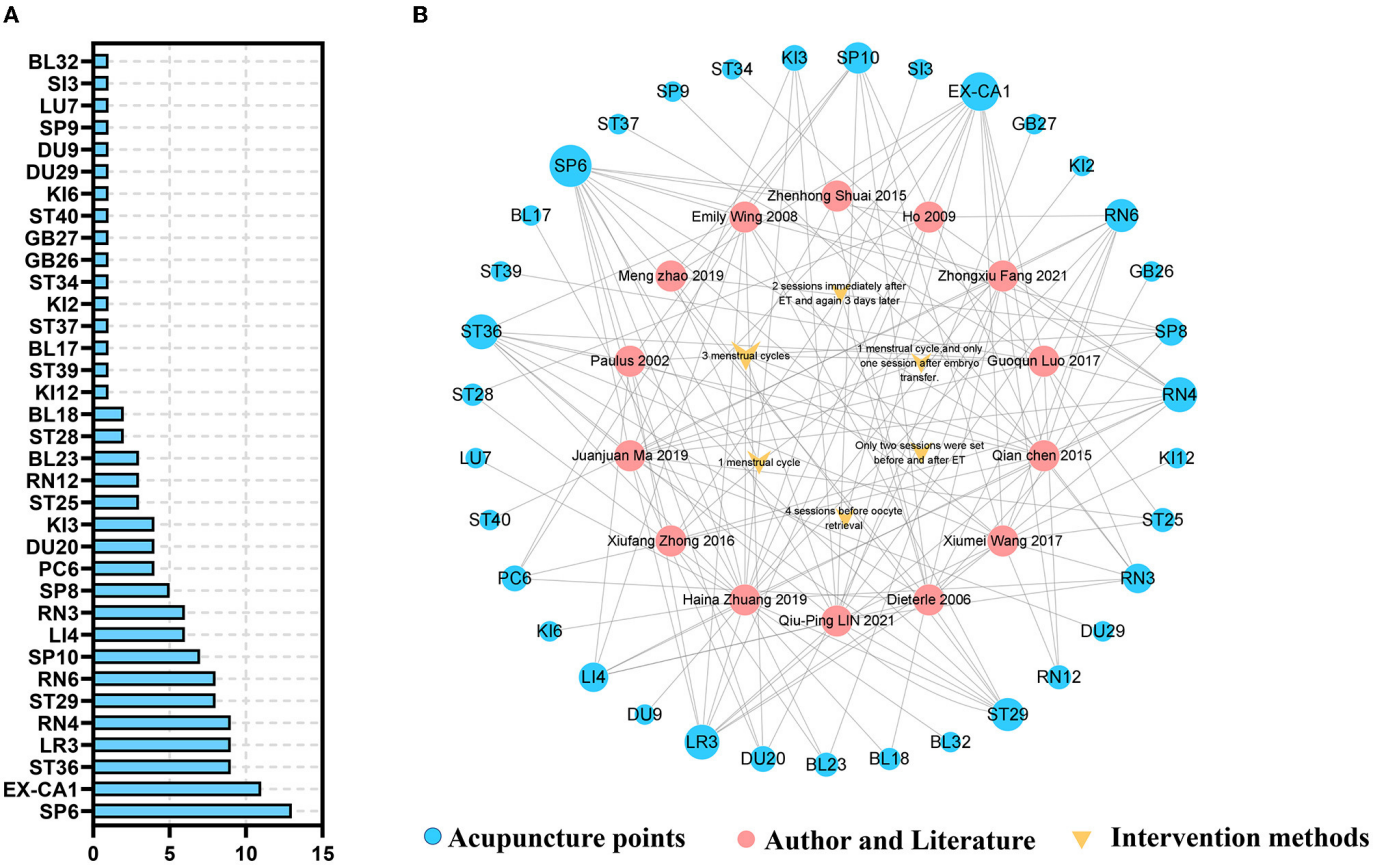
The acupoints and acupuncture duration in included studies. **(A)** The frequency of acupoints in included studies. **(B)** The acupoints-associated and duration-associated network construction (The size of each circle or triangle represent the frequency in the studies).

Besides, the acupoints-associated and duration-associated network construction chart (Figure 2) shows that various acupoints were selected in treating PER. Among the whole trails, SP6, EX-CA1, RE4, ST36, and LR3 were the top five acupoints, and three menstrual cycles were the most frequently used duration for the whole treatment.

### The Methodological Quality of Included Trials

#### Risk Bias of Included Studies

**Figure 3:**
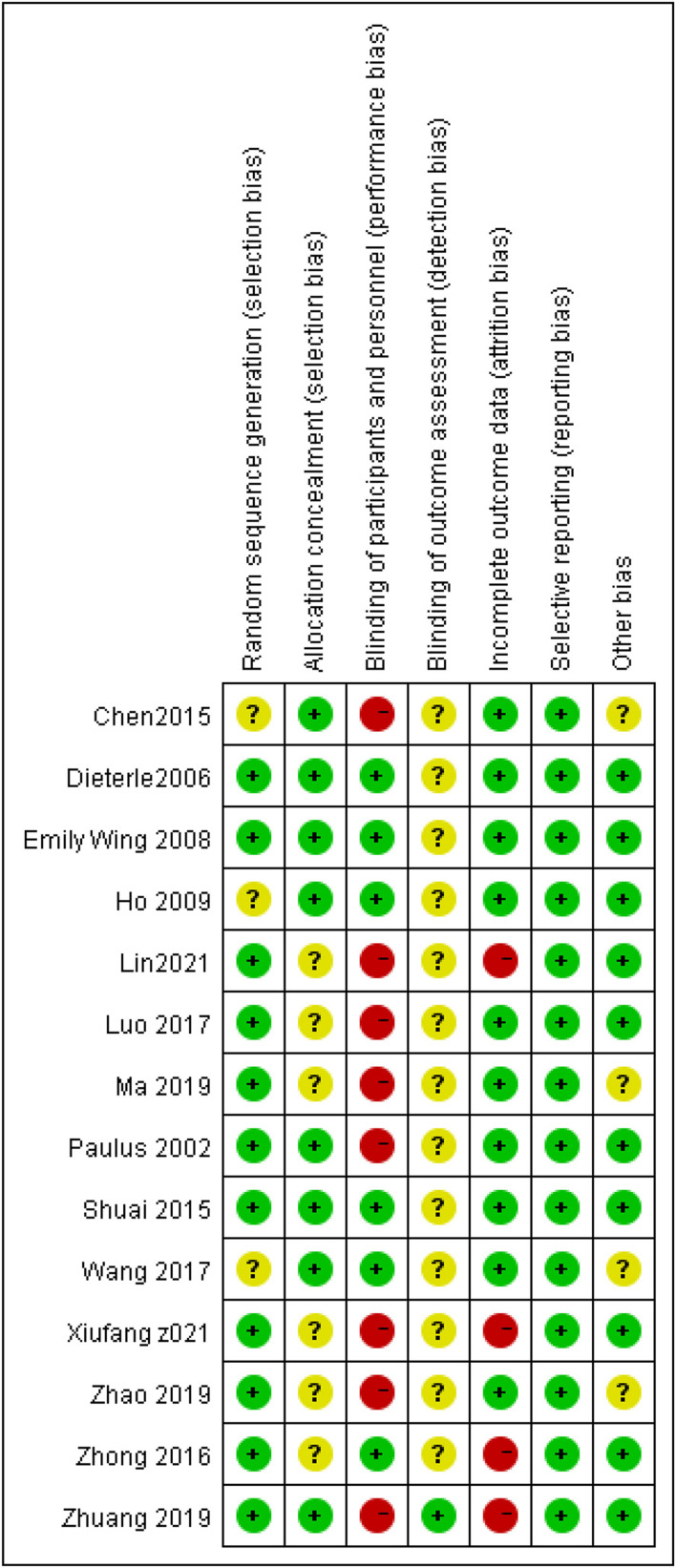
Risk of bias summary of included trials.

A summary of the risks of bias is presented in Table 2 and Figure 3. Three trials (33, 35, 41) did not mention random sequence generation. Eight studies (13, 14, 16, 20, 33, 35, 41, 42) mentioned allocation concealment, which used opaque sealed envelopes subjects to assign included subjects randomly. As presented in Table 1, only one study (16) utilized Streitberger control. Zhao et al. (39) used shallow needles (depth <5 mm) placed on the non-acupoints. Dieterle et al. (42) used an actual needling procedure on acupoints that were designed not to affect fertility, Wang et al. (41) conducted sham acupuncture (acupoint pressing stimulated epidermal irritation slightly without de qi), two studies (14, 40) utilized mock TEAS, and others used no intervention as the control group. For 4 studies (20, 37, 38, 40), some randomized women were recruited from the beginning but did not complete the treatment (i.e., there was no embryo transfer or they did not complete the ER tests), and missing data were not reported which would increase the attrition bias. Four studies (33, 36, 39, 41) remained with other unclear biases.

**Table 2:** Risk of bias summary of included trials.

	**Random sequence generation (selection bias)**	**Allocation concealment (selection bias)**	**Blinding of participants and personnel (performance bias)**	**Blinding of outcome assessment (detection bias)**	**Incomplete outcome data (attrition bias)**	**Selective reporting (reporting bias)**	**Other bias**
So (16)	Low risk	Low risk	Low risk	Unclear risk	Low risk	Low risk	Low risk
Dieterle et al. (42)	Low risk	Low risk	Low risk	Unclear risk	Low risk	Low risk	Low risk
Ho et al. (35)	Unclear risk	Low risk	Low risk	Unclear risk	Low risk	Low risk	Low risk
Paulus et al. (13)	Low risk	Low risk	High risk	Unclear risk	Low risk	Low risk	Low risk
Chen and Hau (33)	Unclear risk	Low risk	High risk	Unclear risk	Low risk	Low risk	Unclear risk
Shuai et al. (14)	Low risk	Low risk	Low risk	Unclear risk	Low risk	Low risk	Low risk
Zhong et al. (43)	Low risk	Unclear risk	Low risk	Unclear risk	High risk^‡^	Low risk	Low risk
Wang et al. (41)	Unclear risk	Low risk	Low risk	Unclear risk	Low risk	Low risk	Unclear risk ^§^
Luo et al. (34)	Low risk	Unclear risk	High risk	Unclear risk	Low risk	Low risk	Low risk
Zhao et al. (39)	Low risk	Unclear risk	High risk	Unclear risk	Low risk	Low risk	Unclear risk^¶^
Ma and Zhang (36)	Low risk	Unclear risk	High risk	Unclear risk	Low risk	Low risk	Unclear risk
Zhuang (20)	Low risk	Low risk	High risk	Low risk	High risk^‡^	Low risk	Low risk
Lin et al. (37)	Low risk	Unclear risk	High risk	Unclear risk	High risk^‡^	Low risk	Low risk
Zhong et al. (38)	Low risk	Unclear risk	High risk	Unclear risk	High risk^‡^	Low risk	Low risk

‡ *In these trials, one to three participants fell out due to interrupt treatment and did not finish the whole treatment, but the authors did not synthesis the intention to treat analysis*;

§ *This trail did not mention about the procedure of IVF-ET, and the measurement of endometrium thickness or endometrium pattern*;

¶ *In this trial, the measurements of ER were not mentioned*.

*We considered the outcomes of ER, especially about the ultrasound procedure might cause measurement bias. But the outcome of CPR would not be expected to cause an important bias due to the measurements. Thus, we thought the bias is not clear*.

#### GRADE for Quality of Each Evidence

Fourteen reviews (13, 14, 16, 20, 33–42) included 24 outcomes that were related to the efficacy of acupuncture for PER. There was low or very low evidence to indicate that acupuncture might improve the CPR or ER parameters when acupuncture was performed in low-, moderate-, or high-dosage groups. The qualities of the evidence are shown in Table 3.

**Table 3:** Quality of evidence-based on GRADE.

**Outcome indicators**	**No. of studies**	**No. of participants**		**Effect [95% CI]**	***I^**2**^***	**Quality of evidence**
		**Intervention group**	**Control group**			
**CPR**						
Low dosage group	4	411	388	1.42 [0.63, 3.20]	82%	⊕○○○ Very low (b, d, e)
Moderate dosage group	4	153	150	2.00 [1.24, 3.22]	0%	⊕⊕○○ Low (a, e)
High dosage group	6	212	220	2.49 [1.67, 3.72]	0%	⊕○○○ Low (a, e)
**Total**	**14**	**776**	**758**	**1.97 [1.97, 2.79]**	**56%**	⊕○○○ Very low (a, b, e)
**EMP**						
Moderate dosage group	3	115	116	1.41 [0.25, 7.90]	85%	⊕○○○ Very low (a, b, d)
High dosage group	6	212	220	3.25 [2.05, 5.15]	10%	⊕⊕○○ Low (a, e)
**Total**	**9**	**327**	**336**	**2.48 [2.26, 4.90]**	**71%**	⊕○○○ Very low (a, b, e)
**LBR**						
Low dosage group	1	185	185	0.68 [0.44, 1.05]	-	⊕○○○ Very low (d, e)
High dosage group	2	68	69	2.96 [1.42, 6.16]	0%	⊕⊕○○ Low (a, e)
**Total**	**3**	**253**	**254**	**1.68 [0.54, 5.27]**	**83%**	⊕○○○ Very low (a, b, d, e)
**EMT**						
Low dosage group	2	196	189	−0.06 [−0.54, 0.41]	81%	⊕○○○ Very low (b, d, e)
Moderate dosage group	4	153	150	0.75 [0.08, 1.42]	87%	⊕○○○ Very low (a, b, e)
High dosage group	5	182	190	0.51 [0.04, 0.98]	80%	⊕○○○ Very low (a, b, e)
**Total**	**11**	**531**	**529**	**0.48 [0.13, 0.83]**	**87%**	⊕⊕○○ Low (a, b)
**RI**						
Moderate dosage group	4	153	150	−0.74 [−1.44,−0.04]	88%	⊕○○○ Very low (a, b, e)
High dosage group	5	185	188	−0.94 [−1.41, −0.48]	77%	⊕○○○ Very low (a, b, e)
**Total**	**9**	**338**	**338**	–**0.86 [**–**1.23**, –**0.48]**	**82%**	⊕○○○ Very low (a, b, e)
**PI**						
Low dosage group	3	140	108	−0.13 [−0.45, 0.20]	23%	⊕⊕○○ Low (d, e)
Moderate dosage group	3	115	116	−1.02 [−1.64, −0.40]	79%	⊕○○○ Very low (a, b, e)
High dosage group	5	185	188	−2.35 [−3.59, −1.11]	96%	⊕○○○ Very low (a, b, e)
**Total**	**11**	**440**	**412**	–**1.33 [-1.93**, –**0.72]**	**93%**	⊕○○○ Very low (a, b, e)
**S/D**						
Moderate dosage group	2	80	81	−2.24 [−3.86, −0.62]	93%	⊕○○○ Very low (a, b, e)
High dosage group	2	89	90	−1.61 [−3.87, 0.65]	97%	⊕○○○ Very low (a, b, e)
**Total**	**4**	**169**	**171**	–**1.91 [**–**3.08**, –**0.75]**	**95%**	⊕○○○ Very low (a, b, e)

*a: Download one level for serious risk of bias: failure to develop and apply appropriate eligibility criteria (inclusion of control population), flawed measurement of both exposure and outcome, failure to adequately control confounding, or Incomplete or inadequately short follow-up. b: Downgraded one level for serious inconsistent: inconsistency refers to an unexplained heterogeneity of results, which includes the wide variance of point estimates across studies, minimal or no overlap of confidence intervals (CI), and statistical criteria, including tests of heterogeneity which test the null hypothesis that all studies have the same underlying magnitude of effect, have a low p-value (p < 0.05), indicating to reject the null hypothesis. c: Downgraded one level for serious indirectness: including differences in the population (applicability), differences in interventions (applicability), differences in outcomes measures (surrogate outcomes), and indirect Comparisons. d: Downgraded one level for serious imprecision: dichotomous outcomes and continuous outcomes were considered separately, including that, optimal information size criterion was not med, or 95% CI overlaps no effect. e: Downgraded one level when publication bias is suspected*.

### Efficacy Analysis

The RCTs included in this study varied in study design, especially in ways of intervention of treatment group, such as, manual acupuncture (MA) alone (16, 36), electro-acupuncture (EA) alone (35, 38, 39), MA or EA with moxibustion (20, 33, 34, 37, 41), MA with auricular acupuncture (13, 42), and TEAS (14, 40). Meanwhile, the duration of acupuncture differed for trails, such as 2 or 4 days of acupuncture, one or three menstrual cycles. Finally, we categorized these trails according to the duration of acupuncture treatment in three groups: the high-dosage group [three menstrual cycles; (14, 20, 33, 34, 37, 41)], the moderate-dosage group [one menstrual cycle; (36, 38–40)], and the low-dosage group [2 or 4 days; (13, 16, 35, 42)].

#### Comparisons of Endometrial Receptivity Outcomes by the Duration of Acupuncture

##### Intervention With Low Dosage

**Figure 4:**
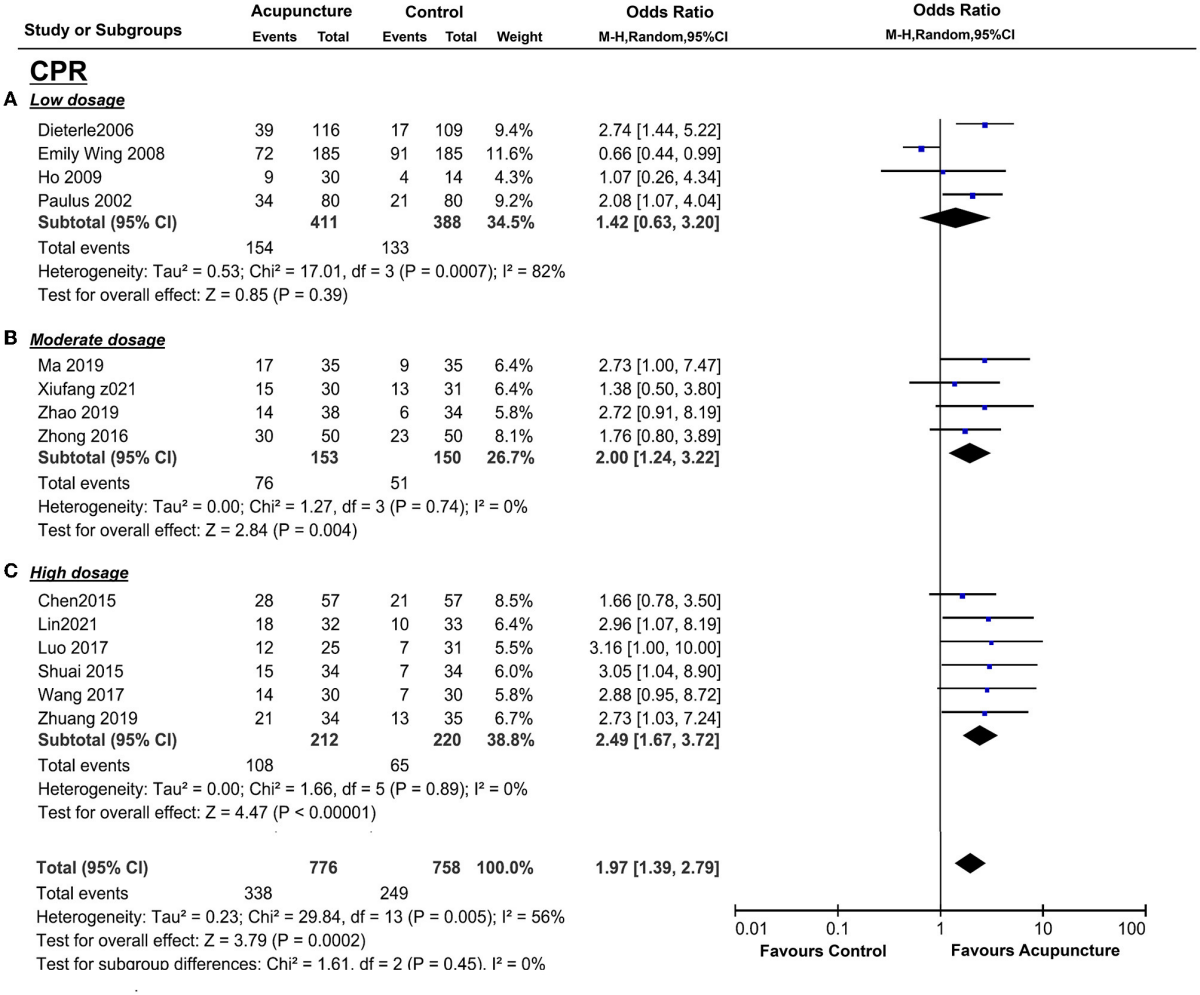
Effects of acupuncture on CPR based on intervention of low dosage **(A)**, moderate dosage **(B)**, and high dosage **(C)**.

For the primary outcome, CPR was from four trials [(13, 16, 35, 42); *n* = 815], but the statistical between the studies was found to be significant (*I*^2^ = 82%, *p* = 0.007). A random-effect model was performed, and no statistical significance was found (*p* = 0.39, OR = 1.42, 95%CI [0.63, 3.20] (Figure 4A).

**Figure 5:**
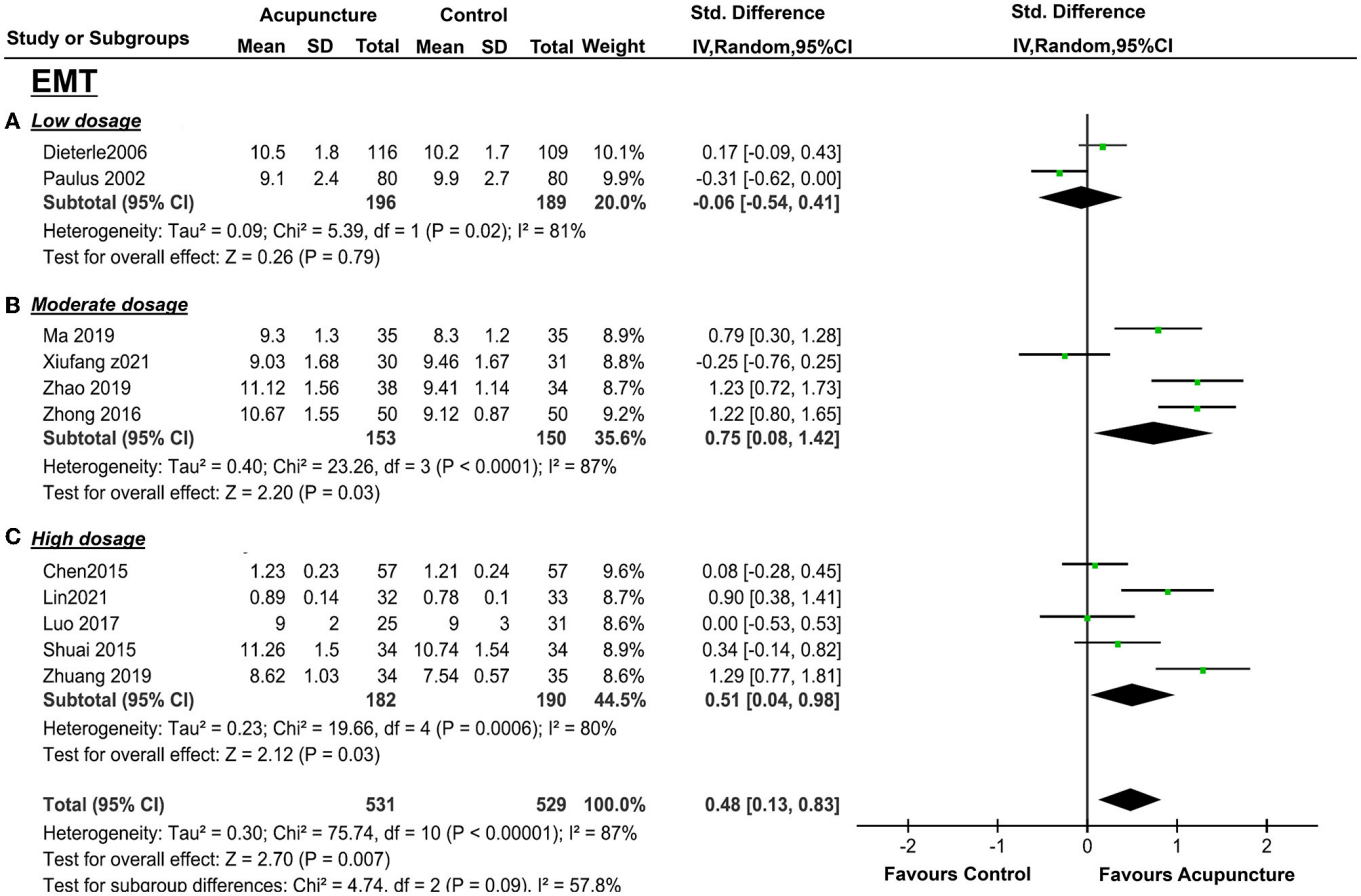
Effects of acupuncture on EMT based on intervention of low dosage **(A)**, moderate dosage **(B)**, and high dosage **(C)**.

**Figure 6:**
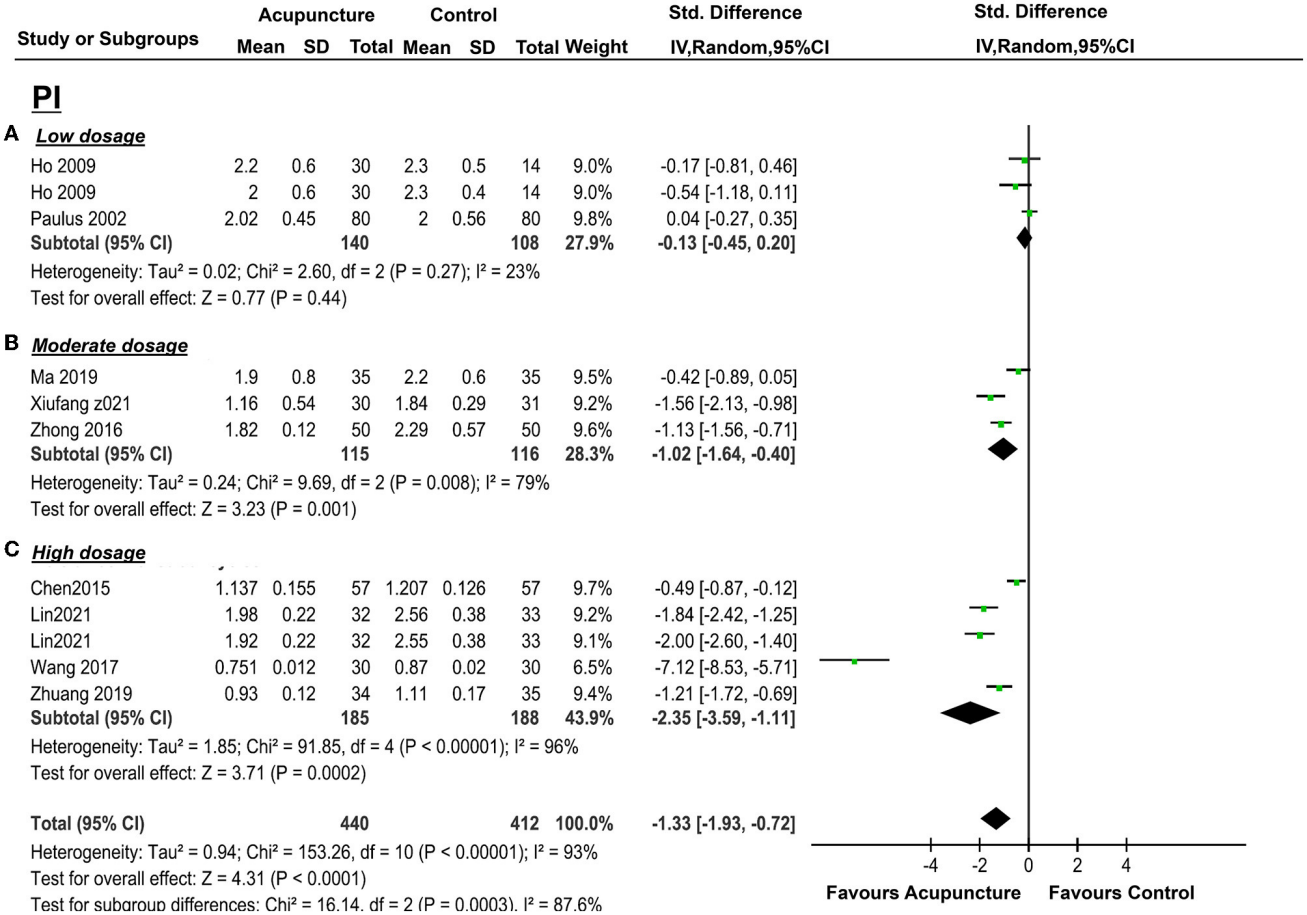
Effects of acupuncture on PI based on intervention of low dosage **(A)**, moderate dosage **(B)**, and high dosage **(C)**.

**Figure 7:**
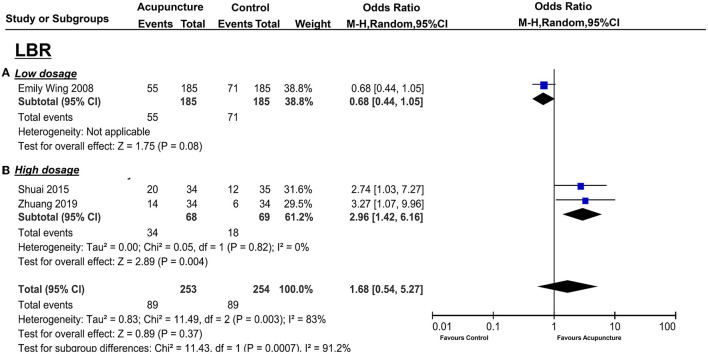
Effects of acupuncture on LBR based on intervention of low dosage **(A)** and high dosage **(B)**.

For the secondary outcomes, EMT was available in two trials (43, 44; *n* = 385), and there was significant heterogeneity between these trials (*I*^2^ = 81%, *P* = 0.02). Pooling the results of these two trials into the random-effects showed no distinct EMT between the treatment group and the control group (*P* = 0.79, SMD = −0.06 95%CI [−0.54, 0.41] (Figure 5A). Only two trials (36, 44; *n* = 160) collected PI, and Ho et al. (35) measured the left and right uterine arteries that were separated into two groups for analysis. No statistical significance was performed (*p* = 0.44, SMD = −0.13, 95%CI [−0.45, 0.20] (Figure 6A), and mild heterogeneity was found between the studies (*I*^2^ = 23%, *p* = 0.27) in the random-effect model. LBR was from only one study (16), and the measurement of acupuncture with the control group did not show a statistically significant benefit (*P* = 0.08, OR = 0.68, 95%CI [0.44, 1.05] (Figure 7A). EMP and RI were not measured with 2 or 4 days of acupuncture.

##### Intervention With Moderate Dosage

Four studies [(36, 38–40); *n* = 309] conducted acupuncture treatment for one menstrual cycle, and CPR data were found in all of these trials. There was significantly higher CPR of acupuncture vs. the control group (*P* = 0.004, OR = 2.00, 95%CI [1.24, 3.22] without heterogeneity (*I*^2^ = 0%, *P* = 0.74; Figure 4B).

**Figure 8:**
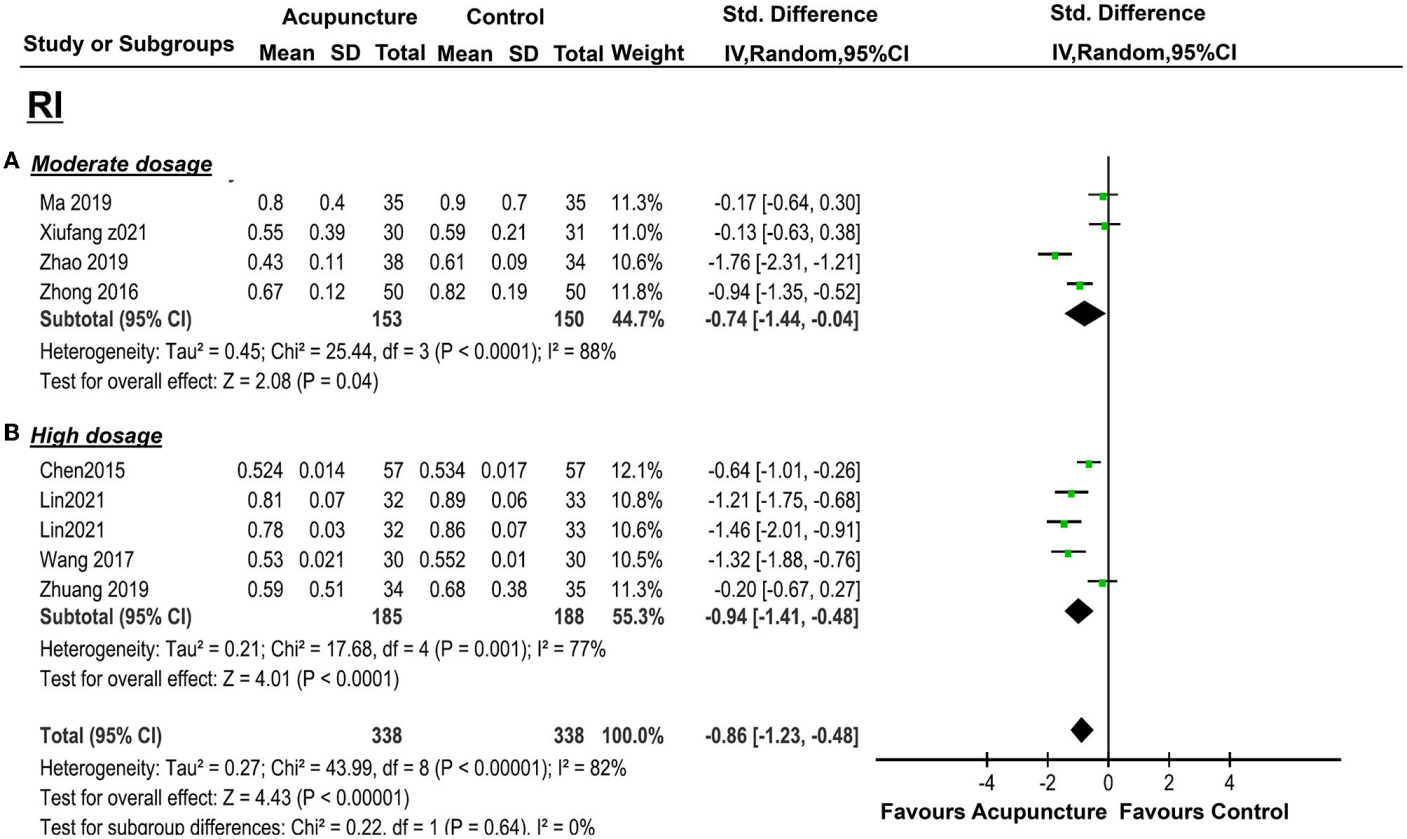
Effects of acupuncture on RI based on intervention of moderate dosage **(A)** and high dosage **(B)**.

**Figure 9:**
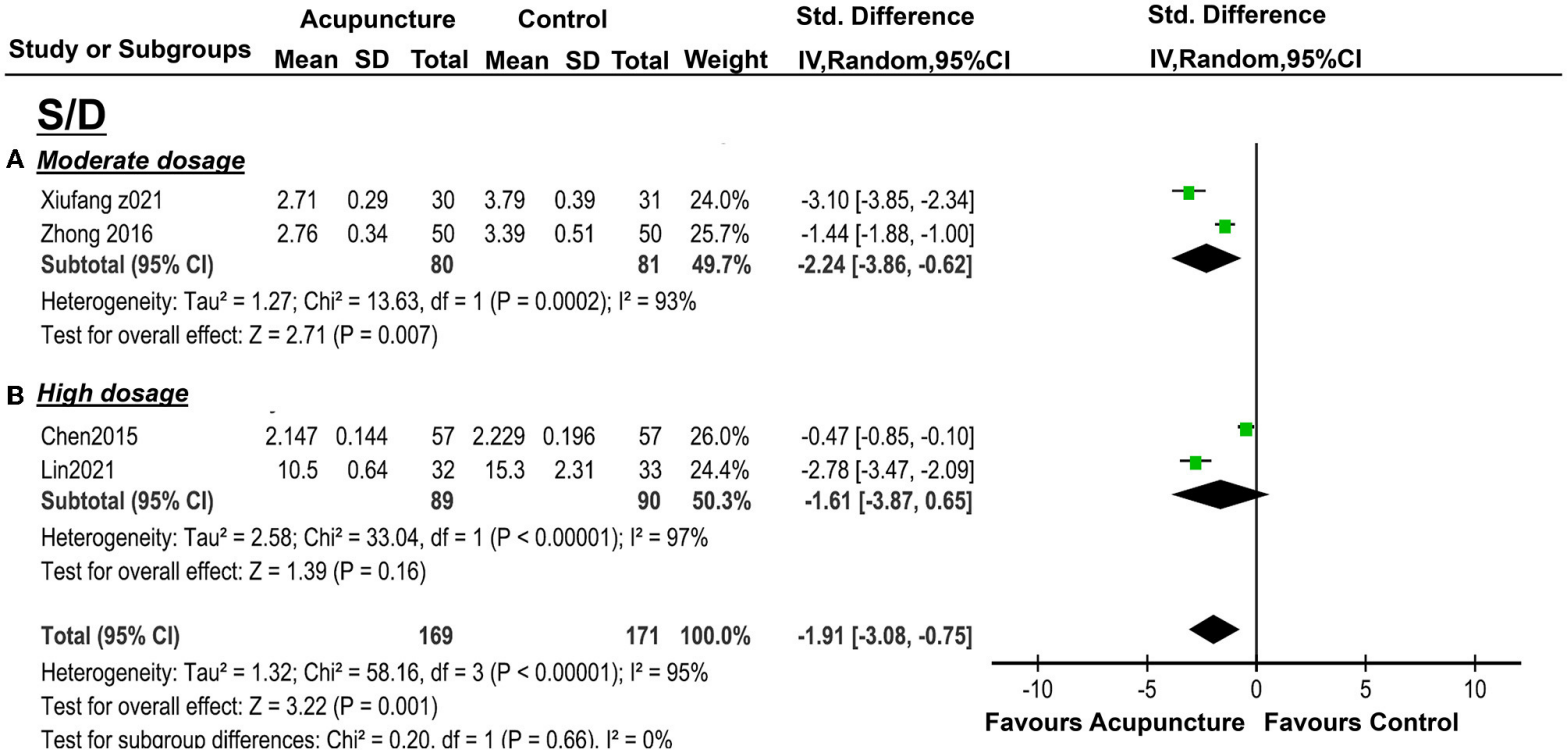
Effects of acupuncture on S/D based on intervention of moderate dosage **(A)** and high dosage **(B)**.

**Figure 10:**
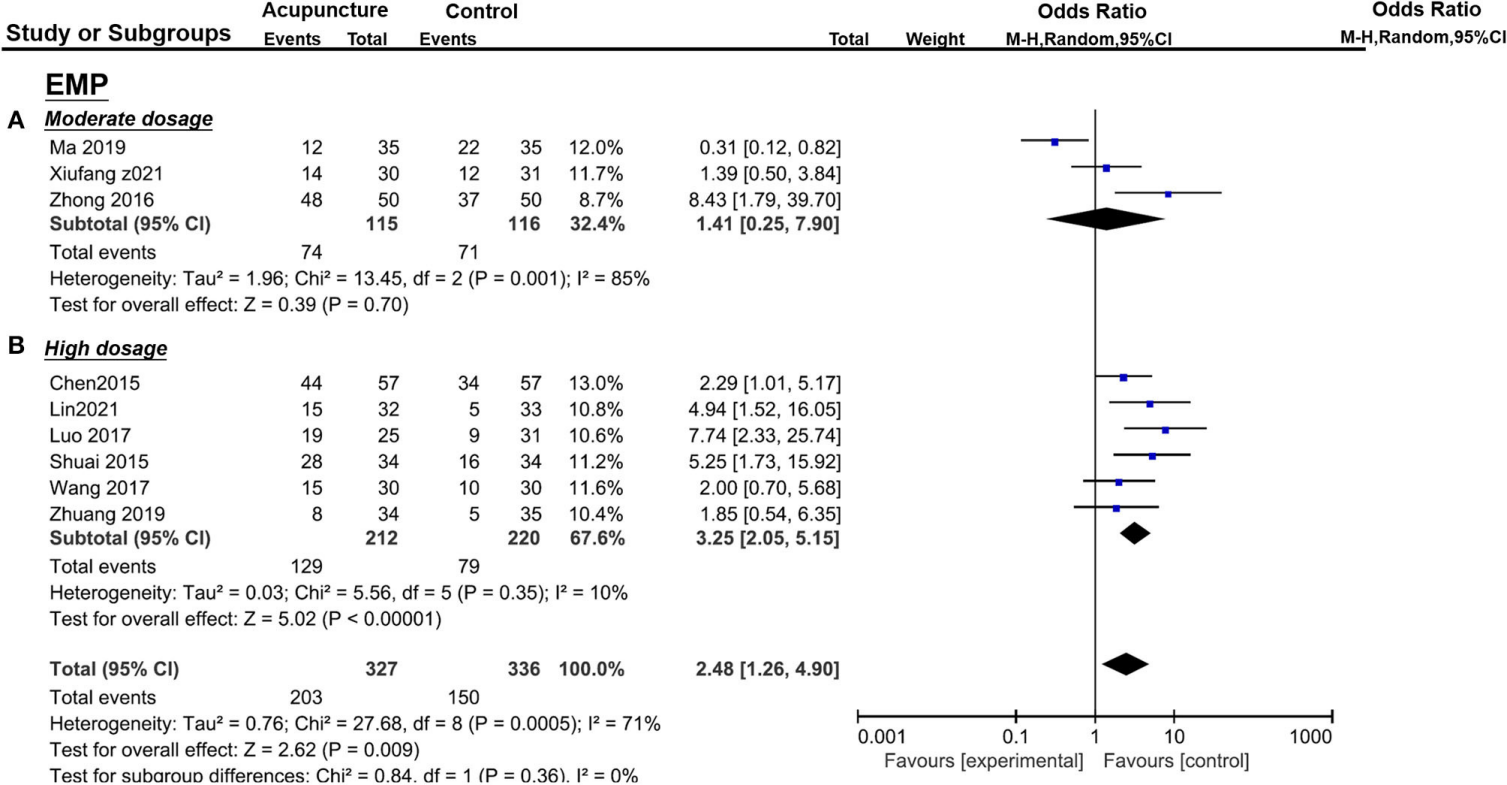
Effects of acupuncture on EMP based on intervention of moderate dosage **(A)** and high dosage **(B)**.

There were also significant benefits in EMT, PI, RI, S/D. A total of four trials [(36, 38, 43, 44); *n* = 309] included EMT, the acupuncture treatment significantly increased the endometrial thickness (*p* = 0.03, SMD = 0.75, 95%CI [0.08, 1.42]) with significant heterogeneity (*I*^2^ = 87%, *p* < 0.01) (Figure 5B); three trails(37, 39, 41; *n* = 237) included PI, acupuncture can significantly decreased PI (*p* = 0.01, SMD = −1.02, 95%CI [−1.64, −0.40] with high heterogeneity (*I*^2^ = 79%, *p* = 0.008) (Figure 6B); four trails (37, 39–41; *n* = 309) conducted RI, acupuncture can significantly reduce the RI (*p* = 0.04, SMD = −0.74, 95%CI [−1.44, −0.04] with high heterogeneity (*I*^2^ = 88%, *p* < 0.01) (Figure 8A); and two trails (39, 41; *n* = 167) conducted S/D, acupuncture can significantly reduce S/D (*P* = 0.007, SMD = −2.24, 95%CI [−3.86, −0.62] with significant heterogeneity (*I*^2^ = 79%, *P* < 0.01) (Figure 9A). No significant difference was found in EMP [three trails (36, 38, 40), *n* = 237, *P* = 0.070, OR = 1.41, 95%CI [0.25, 7.9]; Figure 10A]. LBR was not measured with acupuncture treatment for one menstrual cycle before embryo transfer.

##### Intervention With High Dosage

The pooled indicators of CRR, EMT, PI, EMP, RI, and LBR showed significant differences between the acupuncture group and the control group, while S/D showed non-significant differences. A total of six trails [(14, 20, 33, 34, 37, 41); *n* = 440] evaluated the CPR, acupuncture can significantly improve CPR (*P* < 0.05, OR=2.49, 95%CI [1.67, 3.72] without heterogeneity (*I*^2^ = 0 %, *P* = 0.89) (Figure 4C). For ER parameters, EMT was significantly thicker in the acupuncture group from five trials (14, 20, 33, 34, 37) (*n* = 380, *P* = 0.03, SMD = 0.51, 95%CI [0.04, 0.98] with high heterogeneity (*I*^2^ = 80%, *P* < 0.01; Figure 5C); PI: four trails (20, 33, 37, 41), *n* = 316, *P* < 0.05, SMD = −2.35, 95%CI [– 3.59, −1.11] with high heterogeneity (*I*^2^ = 96%, *P* < 0.01; Figure 6C); RI: four trails (20, 33, 37, 41), *n* = 316, *P* < 0.05, SMD = −0.94,95%CI [−1.41, −0.48] with high heterogeneity (*I*^2^ = 77%, *P* < 0.01; Figure 8B); EMP was significantly improved in 6 trails (14, 20, 33, 34, 37, 41), *n* = 440, *P* < 0.05, OR = 3.25, 95%CI [2.05, 5.15] with small heterogeneity (*I*^2^ = 10%, *P* = 0.35) (Figure 10B). For LBR, it was significantly increased in two trails (14, 20), *n* = 140, *P* = 0.004, OR-2.96, 95%CI [1.42, 6.16]) with non-significant heterogeneity (*I*^2^ = 0%, *P* = 0.82) (Figure 7B). However, there was no significant difference between the acupuncture treatment group and the control group in S/D [two trails (33, 37), *n* = 184, *P* = 0.16, SMD = −1.61, 95%CI (−3.78, 0.65)] with high heterogeneity (*I*^2^ = 97%, *p* < 0.01; Figure 9B).

#### Subgroup Analysis and Investigation of Heterogeneity

However, the duration of acupuncture subgroup analysis did not significantly explain this statistical heterogeneity [interaction *p* = 0.53 for clinical pregnancy outcome and interaction *p* = 0.09 for endometrium thickness, respectively (Figure 4)]. We used subgroup analysis to find out the heterogeneities of the outcomes. With a wide range of study designs, we chose seven variables to assess heterogeneity, according to four clinical characteristics and three methodological characteristics. The heterogeneity analysis was only conducted in EMT, EMP, RI, and PI due to the amounts of included studies.

**Figure 11:**
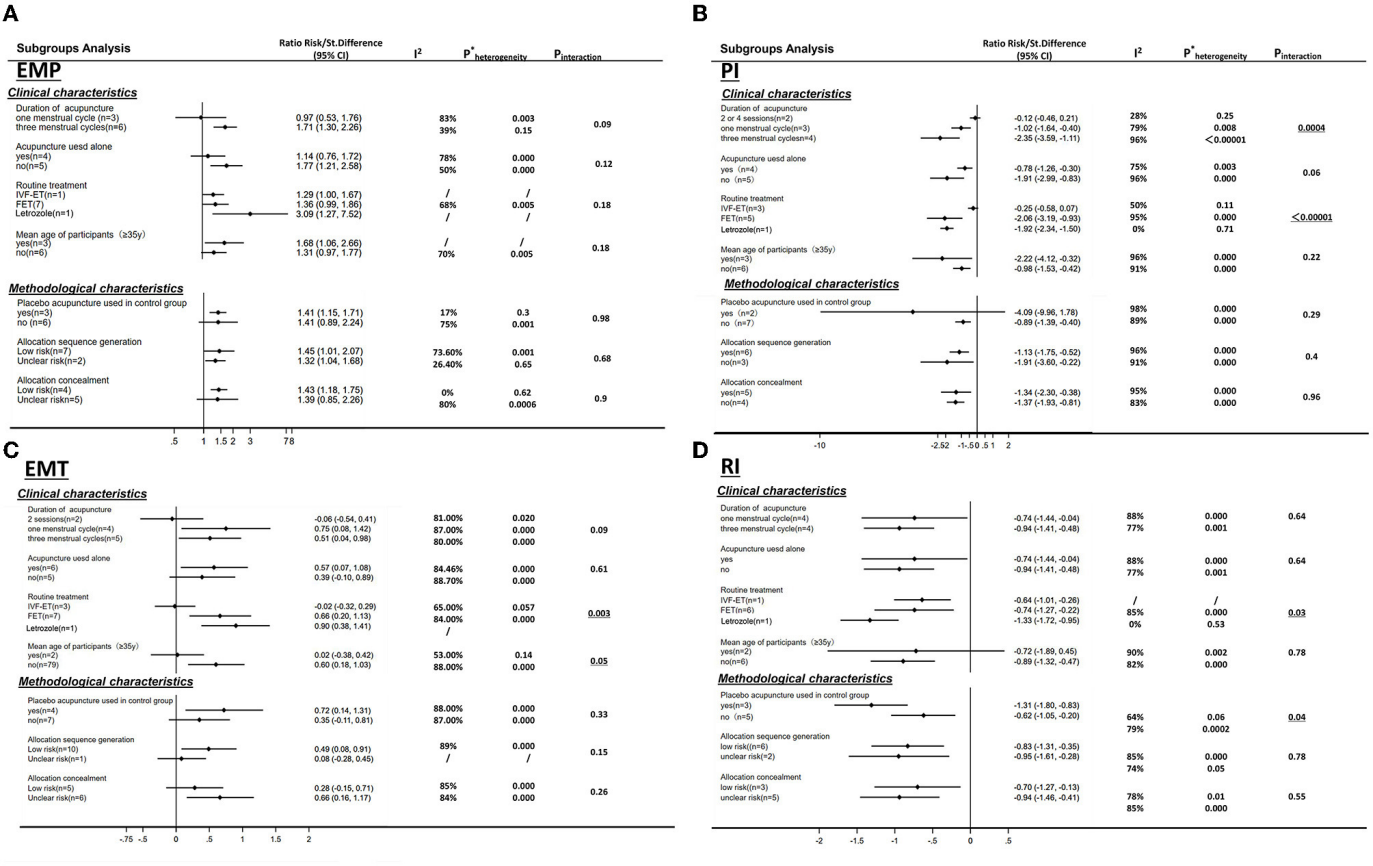
Heterogeneity analysis of four endometrial receptivity characteristics by seven variables. **(A)** endometrial pattern; **(B)** pulse index; **(C)** endometrial thickness; **(D)** resistance index. Seven variables including four clinical characteristics. Acupuncture duration, intervention of different routine treatment (IVF-ET, FET or clomiphene for ovulation induction), acupuncture used alone or with other interventions and mean age of participants (≥35y or not) and three methodological characteristics blinding of participants (sham/placebo or no adjuvant treatment); random sequence generation (adequate or not); allocation concealment (adequate or not) (^*^:0.000 of P_heterogeneity_ means <0.00001).

Generally, 4 subgroup variables (routine treatment, risk of performance bias, duration of acupuncture treatment, and the age of participants) could explain some of the heterogeneities across all trials (Figure 11). Routine treatment was significantly different with subgroup analysis in EMT, RI, PI (EMT: interaction *p* = 0.003; RI: interaction *p* = 0.03; PI: interaction *p* < 0.05, respectively), risk of performance bias (blinding of participants and personnel) was significantly different with subgroup analysis in RI (interaction *P* = 0.003). Subgroup analysis in PI was significantly different when restricted in the duration of acupuncture treatment (*P* = 0.004), and EMT was almost statistically significant when restricted in the age of participants (interaction *P*=0.05). That is, heterogeneity is reduced when restricted to variables (i.e., the maximum *I*^2^ percentage change of the subgroup was 43%).

Furthermore, considering the controlled intervention of different comparisons is the heterogeneity, and we made a further subgroup analysis in different dosage groups according to the different way of controlled group: no intervention or placebo treatment. As shown in Supplementary Material S3, though we found converse results in the re-subgroup analysis, the heterogeneities remained high among the groups.

#### Publication Bias Analysis

**Figure 12:**
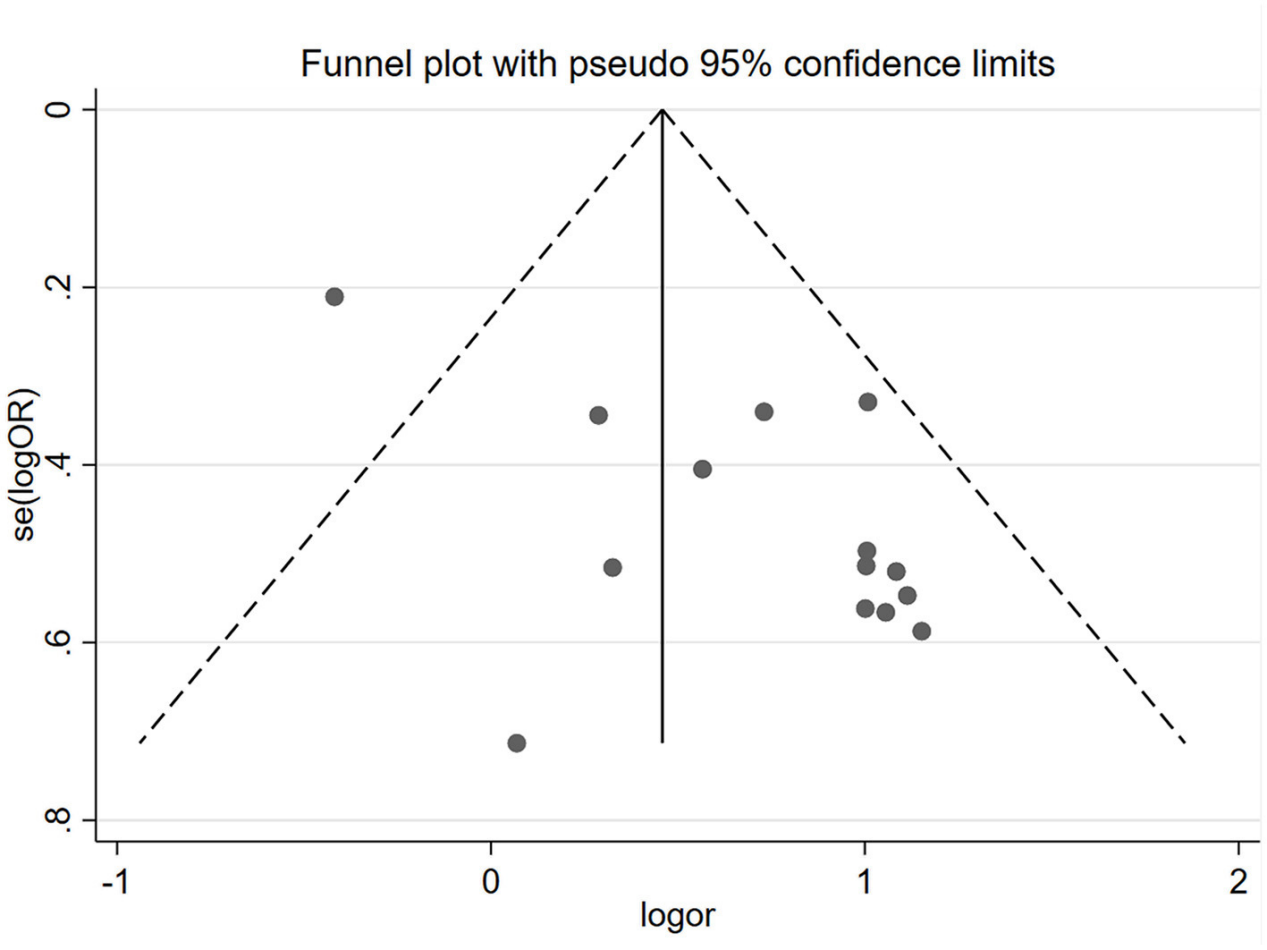
Funnel plot of trials meeting inclusion criteria (CPR). The Egger'test *P* = 0.003.

The funnel plot (see Figure 12) of the primary outcome CPR showed a small positive studies effect, with the smaller studies showing more benefit being conducted than larger studies, and also positive results were easily published by researchers. The Eggers'test *p* = 0.003, which means there is public bias in the collected studies.

## Discussions

### Summary of the Main Results

To the best of our knowledge, this is the first comprehensive review addressing the pooled analysis of whether dose-related acupuncture therapeutic efficacy improves ER for infertile women. Finally, we collected 14 trials in our review, including four (36–39) updated relevant studies.

Generally, acupuncture can significantly improve the CPR and the ER parameters (EMT, EMP, RI, PI, and S/D) with moderate or high heterogeneity (*I*^2^: 56–95%), whereas the LBR was not statistically significant between the two groups with high heterogeneity (*I*^2^ = 83%), also. For the primary outcome, CPR was significantly increased in the moderate and high acupuncture groups without heterogeneity, whereas in the low-dosage group, CPR failed to show a significant improvement with considerable heterogeneity (*I*^2^ = 82%) (Table 3). For the ER parameters, EMP was significantly improved in the high-dosage group with a small heterogeneity (*I*^2^= 10%) and was not statistically significant in the moderate group with considerable heterogeneity (*I*^2^ = 85%) (Table 3). Though the total heterogeneity of EMP was moderate (*I*^2^ = 71%), we failed to find the sources of the heterogeneity (Figure 11A). The decrease of PI showed a significant difference in the high-dosage and moderate group between the acupuncture group vs. the controlled one with considerable heterogeneity (*I*^2^ = 96% and *I*^2^ = 79%, respectively), whereas, in the low-dosage group, no significant difference was found with considerable heterogeneity (Table 3). The total heterogeneity of PI was high (*I*^2^ = 93%) in our analysis, and not only the duration of acupuncture but also the different routine treatments for infertile women were attributed to the source of heterogeneity (interaction *P* < 0.01, <0.01, respectively) (Figure 11B). EMT was significantly improved in the moderate- and high-dosage groups with significant heterogeneity (*I*^2^ = 87%, *I*^2^ = 80%, respectively), and was not statistically different in the low-dosage group with considerate heterogeneity (*I*^2^ = 81%). The total heterogeneity of EMT was high (*I*^2^ = 87%) too, and we found out that the different routine treatment was attributed to the source of heterogeneity (interaction *P* = 0.003). The heterogeneity was decreased and was 65% in the IVF-ET group and 84% in the FET group (Figure 11C). Though the significant decreases of RI were shown in the moderate- and high-dosage acupuncture groups, the heterogeneities were significant too (*I*^2^ = 88%, *I*^2^ = 77%, respectively) (Table 3). In our subgroup analysis, the routine treatment and the method of the compared intervention (placebo acupuncture was utilized or not) may be the sources of heterogeneity (Figure 11D). LBR was statistically improved in the high-dosage group while it was non-significant in the low-dosage group. S/D was significantly decreased in both moderate and high-dosage groups. However, the indicators of S/D and LBR showed high heterogeneities in the studies (*I*^2^ = 95, *I*^2^ = 83%, respectively) (Table 3). Considering the small numbers of included studies that measured the LBR and the S/D, the heterogeneity analysis was not performed. Above all, comparisons were evaluated as a very low to moderate level of evidence, even very low and low, mostly based on the GRADE (Table 3).

Thus, the therapeutic effect of acupuncture in different dosage groups is relatively weak so far. Though it is difficult to draw a definitive conclusion that acupuncture is more effective, we discussed the findings based on our subgroup analysis under the method of study design.

### Analyses Based on Acupuncture Dosage

Above all, the evaluation among the outcomes from the included studies and acupuncture dosage resulted in different therapeutic effects. Though significant heterogeneities were not found, the trend of relatively more dosage showed better effects in CPR, EMT, PI (moderate or high dosage showed significantly improved while the low dosage did not), and EMP (high dosage was significantly improved while the moderate did not; Table 3).

Our findings of the relationship between acupuncture therapeutic efficacy and the dosage were consistent with the previous studies. White et al.(45) thought the dose may be affected by the state of the patient (e.g., nervous, immune, and endocrine systems); different doses may be required for different conditions. For the analgesia of acupuncture, a system review (46) evaluated whether the effect of acupuncture is dose-related for symptom management in knee osteoarthritis. Based on the consideration of the number of points needled during each treatment; de qi response; frequency of treatment per week; and several treatment sessions, the study found out that the effect may be associated with the dose of acupuncture, with a higher dosage (the number of points needled ≥9; de qi response; frequency ≥2 sessions a week; the total number of treatment sessions ≥8) related to better treatment outcomes in terms of relief of pain and dysfunction in patients with knee osteoarthritis. Gao (47) utilized functional MRI (fMRI) in evaluating acupuncture efficacy in treating migraine. Four weeks of acupuncture treatments strengthened the brain connection to improve therapeutic efficacy in migraine when compared with 2 weeks. For the acupuncture modulation in default mode network (DMN) of the brain with fMRI, Lin et al. (48) suggested that enhancing the acupuncture dose could potentially be applied as a means of modulating brain activity. For improving the outcome of women who underwent IVF, Magarelli et al.(12) suggested that patients receiving acupuncture weeks before the stimulation of IVF (usually 4 weeks at two treatments per week until the day of retrieval) and in the times before egg retrieval and ET may benefit more than those patients treated just pre-/post-ET. In a systematic and meta-review, Zheng et al. (49) reported that the pooled biochemical pregnancy rate (BPR) was significantly higher in the acupuncture group than in the controls (OR = 2.07, *P* = 0.02). The CPR, LBR, and IR results tended to be higher when acupuncture treatment was conducted during the COH time (from the starting time of the menstrual cycle to the oocyte retrieval). Whereas, the pooled BPR, CPR, OPR, LBR, IR, and MR showed no significant differences when acupuncture was performed only around the day of ET (i.e., CPR: *p* = 0.48).

Infertility for women with PER is a complex medical issue with multiple factors, often associated with previous gynecological issues/pathology (e.g., fibroids, endometriosis, autoimmune disease, etc.). Although patients may benefit from two sessions of acupuncture treatment (13), many studies (15–17, 33) failed to repeat this outcome. Thus, chronic conditions of long-term duration need larger dosages of acupuncture, especially in patients with multiple health issues.

Why S/D is lower in three-cycle treatments than in one cycle? It was found that there were more stressful and uncomfortable feelings during the IVF process in the acupuncture group (50). Also, the S/D is a parameter of the uterine artery. The S/D of women will change under too much stress and discomfort. Besides, how many acupuncture sessions should be performed for participants who suffered PER is necessary to reconsider because of the financial burden and time cost for PER (51).

### Analyses Based on the Cause of Heterogeneity

Heterogeneity was expected due to the variables of the routine treatment, acupuncture treatment duration, performance bias (blinding of participants and personnel), and the age of participants.

For routine treatment for infertile women, to the best of our knowledge, IVF-ET is a procedure in which fresh embryos would be transferred after ovarian stimulation, while FET is frozen embryo transfer. Ovarian stimulation for IVF would affect the luteal phase and the window of implantation (WOI) by altering the endocrinological environment of the endometrium. That is, ovarian stimulation will reduce ER in IVF-ET (52), whereas FET in subsequent cycles avoids possible adverse effects without ovarian stimulation. For women with PCOS, LE has been used as the traditional medication to induce ovulation for being superior to CC in ER (53). But it is uncertain whether LE for PCOS will improve the EMT compared to the programmed COH cycle (54).

For blinding of participants and personnel, placebo acupuncture treatment is not inert in numerous studies (55), and the possibility that some of the sham interventions may have acupuncture-specific efficacy (56, 57). Even in Madsen's review (58), a greater effect of acupuncture with penetrative placebo needles compared with the non-penetrative placebo needles was found (*P* = 0.04). Furthermore, the device or method of different placebo interventions is possible resource of heterogeneity. To the best of our knowledge, four different types of placebo acupuncture: i) placebo acupuncture device (with a blunt needle tip, pressing the skin without penetrating), including Streitberger's needle [(15, 16, 59); the needle tip is blunt but a pricking sensation is felt by the patient, simulating the puncturing of the skin] and Park needle [(60, 61); this device has a retractable needle shaft and a blunt tip. Acupuncturists were instructed to lightly place the sham needle on the surface of the skin with no manipulation of the needle to minimize any physiological effect]; ii)shallow acupuncture (39), which stimulates at the real acupoints (sham acupuncture was standardized as minimal-depth needling without stimulation “DeQi”); iii) non-acupoints (17) or non-therapeutic acupoints (42) utilization in the control group, and iv)Mock electrical stimulation [(14, 62); the technique has been shown to constitute a successful placebo treatment in functional brain networks (61). However, the difference of placebo efficacy among the above placebo intervention was not found in an accepted way (63). Thus, the potential heterogeneity may be also related to the placebo intervention.

For the age of participants, one of the major factors related to the decline in fertility with age is the aging uterus. However, the underlying factors that decrease endometrial receptivity in older women are still unclear.

Though different interventions in the treatment group (acupuncture was used alone or not) showed no significant heterogeneity, the previous meta-analysis showed the intervention in the treatment group accounts for some heterogeneity (64). Further analysis should be conducted. Furthermore, other potential variables, such as the way of measurement of three-dimensional ultrasound, may cause heterogeneity and care should be taken when the therapeutic efficacy of acupuncture treatment is evaluated.

### Research Strengths

To reduce the risk of bias, we only included the studies of randomized trials. We assessed the GRADE score to determine the strength of evidence. We excluded the RCTs with the outcome of EMT only for a more reliable result. To strengthen the efficacy of our findings, we explored the cause of heterogeneity associated with the clinical characteristics and methodological characteristics.

### Research Limitations

The review included moderate or low-quality studies. In our review, the duration of acupuncture was assessed. However, based on the study design of acupuncture trial guideline, Standards for Reporting Interventions in Controlled Trials of Acupuncture (STRICTA), and the frequency and starting time of acupuncture should be also considered in acupuncture dosage evaluation. Also, the variables subgroup analysis did not decrease the heterogeneity, which indicated that there were still potential factors in the collected studies. Therefore, a standardized individualized acupuncture program with a homogeneity trial design needed to be performed to evaluate the therapeutic efficacy of acupuncture, such as enough fixed acupuncture treatment dosage. The latest RCTs or negative unpublished trials were unlikely to be included in our analysis, so there was some publication bias.

## Conclusion

Overall, the trend of relatively higher acupuncture dosage showed better effects for poor endometrial receptivity, though, it is difficult to draw a definitive conclusion with the considered heterogeneities among the outcomes and insufficiently high quality of the evidence. More high-quality studies with a homogeneity trial design need to be conducted to explore the efficacy of acupuncture for improving PER for infertile women. Also, there should be more consensus and dimensionality in the assessment of the measurement of the three-dimensional ultrasound.

## Data Availability Statement

The datasets presented in this study can be found in online repositories. The names of the repository/repositories and accession number(s) can be found in the article/Supplementary Material.

## Author Contributions

XZ conceived and designed the study and wrote the paper. HaY and LL contributed to the literature search and extracted data and conducted the format and tables. XZ and HoY performed the statistical analysis. FW and SY participated in critical dialogue and revised the manuscript. Moreover, all authors have approved the final manuscript for submission.

## Funding

This study was funded by a program of the Science and Technology foundation of Sichuan Province (grant number 2020JDJQ0051).

## Conflict of Interest

The authors declare that the research was conducted in the absence of any commercial or financial relationships that could be construed as a potential conflict of interest.

## Publisher's Note

All claims expressed in this article are solely those of the authors and do not necessarily represent those of their affiliated organizations, or those of the publisher, the editors and the reviewers. Any product that may be evaluated in this article, or claim that may be made by its manufacturer, is not guaranteed or endorsed by the publisher.
