# Supplementary material for: The Dose-Related Efficacy of Acupuncture on Endometrial Receptivity in Infertile Women: A Systematic Review and Meta-Analysis
Source: Front Public Health. 2022 Apr 28;10:858587. doi: 10.3389/fpubh.2022.858587 (PMC9095926; doi:10.3389/fpubh.2022.858587)
Supplement: Supplementary file 2 [file Data_Sheet_2.docx]

## Supplementary 2 Search Strategies

**PubMed search strategy (Feb 25, 2022)=42**

1. **(((((infertility [MeSH Terms]) OR (Sterility, Reproductive [Title/Abstract])) OR (Sterility[Title/Abstract])) OR (Reproductive Sterility[Title/Abstract])) OR (Subfertility[Title/Abstract])) OR (Sub-Fertility[Title/Abstract])**
2. **(((Fertilization in Vitro [MeSH Terms]) OR (Sperm Injections, Intracytoplasmic [MeSH Terms])) OR (Oocyte Retrieval [MeSH Terms])) OR (Ovarian Follicle [MeSH Terms])**
3. **(((((((((((((((((((In Vitro Fertilization[Title/Abstract]) OR (In Vitro Fertilizations[Title/Abstract])) OR (Test-Tube Fertilization[Title/Abstract])) OR (Fertilization, Test-Tube[Title/Abstract])) OR (Fertilizations, Test-Tube[Title/Abstract])) OR (Test Tube Fertilization[Title/Abstract])) OR (Test-Tube Fertilizations[Title/Abstract])) OR (Fertilizations in Vitro[Title/Abstract])) OR (Test-Tube Babies[Title/Abstract])) OR (Babies, Test-Tube[Title/Abstract])) OR (Baby, Test-Tube[Title/Abstract])) OR (Test Tube Babies[Title/Abstract])) OR (Test-Tube Baby[Title/Abstract])) OR (Injection, Intracytoplasmic Sperm[Title/Abstract])) OR (Injections, Intracytoplasmic Sperm[Title/Abstract])) OR (Intracytoplasmic Sperm Injection[Title/Abstract])) OR (Sperm Injection, Intracytoplasmic[Title/Abstract])) OR (Intracytoplasmic Sperm Injections[Title/Abstract])) OR (ICSI[Title/Abstract])) OR (Injections, Sperm, Intracytoplasmic[Title/Abstract])**
4. **((((intracytoplasmic sperm injection*[Title/Abstract]) OR (artificial insemination*[Title/Abstract])) OR (assisted reproduct*[Title/Abstract])) OR (ovulation induc*[Title/Abstract])) OR (infertil*[Title/Abstract])**
5. **#1 OR #2 OR #3 OR #4**
6. **(((((acupuncture [MeSH Terms]) OR (Acupuncture Therapy [MeSH Terms])) OR (Acupuncture Points [MeSH Terms])) OR (Acupressure [MeSH Terms])) OR (Electroacupuncture [MeSH Terms])) OR (Moxibustion [MeSH Terms])**
7. **(((((meridian[MeSH Terms]) OR (meridian*[Title/Abstract])) OR (Ching Lo[Title/Abstract])) OR (Jing Luo[Title/Abstract])) OR (Luo, Jing[Title/Abstract])) OR (Jingluo[Title/Abstract])**
8. (((((((((((((((((((Pharmacopuncture [Title/Abstract]) OR (Acupuncture Treatment [Title/Abstract])) OR (Acupuncture Treatments [Title/Abstract])) OR (Treatment, Acupuncture[Title/Abstract])) OR (Therapy, Acupuncture[Title/Abstract])) OR (Pharmacoacupuncture Treatment [Title/Abstract])) OR (Treatment, Pharmacoacupuncture [Title/Abstract])) OR (Pharmacoacupuncture Therapy [Title/Abstract])) OR (Therapy, Pharmacoacupuncture [Title/Abstract])) OR (Acupotomy [Title/Abstract])) OR (Acupotomies [Title/Abstract])) OR (Acupuncture Point [Title/Abstract])) OR (Point, Acupuncture [Title/Abstract])) OR (Points, Acupuncture [Title/Abstract])) OR (Acupoints [Title/Abstract])) OR (Acupoint [Title/Abstract])) OR (Shiatsu [Title/Abstract])) OR (Zhi Ya [Title/Abstract])) OR (Chih Ya [Title/Abstract])) OR (Shiatzu [Title/Abstract])
9. ((acup*?point*[Title/Abstract]) OR (acup*[Title/Abstract])) OR (trigger adj3 point*[Title/Abstract])
10. ((((((((((((((((((((Transcutaneous Electric Nerve Stimulation[MeSH Terms]) OR (Electric Stimulation, Transcutaneous[Title/Abstract])) OR (transcutaneous electr* stimulat*[Title/Abstract])) OR (nerve stimulat*[Title/Abstract])) OR (electro‐acupuncture[Title/Abstract])) OR (electroacupuncture[Title/Abstract])) OR (neuro‐modulation[Title/Abstract])) OR (neuromodulation[Title/Abstract])) OR (trans‐abdominal stimulat*[Title/Abstract])) OR (sacral nerve stimulat*[Title/Abstract])) OR (interferential electr* stimulat*[Title/Abstract])) OR (Electric Stimulation, Transcutaneous[Title/Abstract])) OR (Electric Stimulation, Transcutaneous[Title/Abstract])) OR (Percutaneous Electrical Nerve Stimulation[Title/Abstract])) OR (Transcutaneous Electrical Nerve Stimulation[Title/Abstract])) OR (Transcutaneous Nerve Stimulation[Title/Abstract])) OR (Nerve Stimulation, Transcutaneous[Title/Abstract])) OR (TENS[Title/Abstract])) OR (Percutaneous Neuromodulation Therapy[Title/Abstract])) OR (Neuromodulation Therapy, Percutaneous[Title/Abstract])) OR (Neuromodulation*, Percutaneous Electrical[Title/Abstract])
11. #5 OR #6 OR #7 OR #8 OR #9 OR #10
12. ((endometrium[Title/Abstract]) OR (uterus[MeSH Terms])) OR (uterine arteries[MeSH Terms])
13. ((((((((((((((((((((((((((Endometria [Title/Abstract]) OR (Endometria receptivity [Title/Abstract])) OR (Uteri [Title/Abstract])) OR (Womb [Title/Abstract])) OR (Wombs [Title/Abstract])) OR (Uterus Cornua [Title/Abstract])) OR (Uterine Cornua [Title/Abstract])) OR (Cornua, Uterine [Title/Abstract])) OR (Uterine Fundus [Title/Abstract])) OR (Fundus, Uterine [Title/Abstract])) OR (Fundus Uteri [Title/Abstract])) OR (Fundus Uterus [Title/Abstract])) OR (Uteri, Fundus [Title/Abstract])) OR (thin endometrium [Title/Abstract])) OR (endometrium thickness [Title/Abstract])) OR (endometrium pattern [Title/Abstract])) OR (Arteries, Uterine [Title/Abstract])) OR (Artery, Uterine [Title/Abstract])) OR (Uterine Arteries [Title/Abstract])) OR (endometrium blood flow [Title/Abstract])) OR (pulse index [Title/Abstract])) OR (resistive index [Title/Abstract])) OR (peak systolic velocity/ end-diastolic blood velocity (S/D) [All Fields])) OR (pulse index[Title/Abstract])) OR (resistive index[Title/Abstract])) OR ("uterine artery"[Title/Abstract])) OR ("uterine blood flow"[Title/Abstract])
14. (endometri*[Title/Abstract]) OR (uter*[Title/Abstract])
15. #12 OR #13 OR #14
16. ((random allocation [MeSH Terms]) OR (clinical trials as topic [MeSH Terms])) OR (randomized controlled trial [Publication Type])
17. #5 AND #11 AND #15 AND #16

**Cocharne search strategy (Feb 25, 2022) =65**

#1 MeSH descriptor: [Infertility] explode all trees

#2 (Sterility, Reproductive):ti,ab,kw OR (Sterility):ti,ab,kw OR (Reproductive Sterility):ti,ab,kw OR (Subfertility):ti,ab,kw AND (Sub-Fertility):ti,ab,kw (Word variations have been searched)

#3 (vitro fertili?ation):ti,ab,kw OR (intrauterine insemination*):ti,ab,kw OR (artificial insemination*):ti,ab,kw OR (assisted reproduct*):ti,ab,kw OR (infertil* or subfertil*):ti,ab,kw

#4 MeSH descriptor: [Acupuncture] explode all trees

#5 MeSH descriptor: [Acupuncture Points] explode all trees

#6 MeSH descriptor: [Acupuncture Therapy] explode all trees

#7 MeSH descriptor: [Acupuncture Therapy] explode all trees

#8 (Pharmacopuncture):ti,ab,kw OR (Acupuncture Treatment):ti,ab,kw OR (Acupuncture Treatments):ti,ab,kw OR (Treatment, Acupuncture):ti,ab,kw OR (Therapy, Acupuncture):ti,ab,kw (Word variations have been searched)

#9 (Pharmacoacupuncture Treatment):ti,ab,kw OR (Treatment, Pharmacoacupuncture):ti,ab,kw OR (Pharmacoacupuncture Therapy):ti,ab,kw OR (Therapy, Pharmacoacupuncture):ti,ab,kw OR (Acupotomy):ti,ab,kw (Word variations have been searched)

#10 (Acupotomies):ti,ab,kw OR (Acupuncture Point):ti,ab,kw OR (Point, Acupuncture):ti,ab,kw OR (Points, Acupuncture):ti,ab,kw OR (Acupoints):ti,ab,kw (Word variations have been searched)

#11 (Acupoint):ti,ab,kw OR (Shiatsu):ti,ab,kw OR (Zhi Ya):ti,ab,kw OR (Chih Ya):ti,ab,kw AND (Shiatzu):ti,ab,kw (Word variations have been searched)

#12 (acup*?point*):ti,ab,kw OR (acup*):ti,ab,kw OR (trigger adj3 point*):ti,ab,kw

#13 ("nerve stimulat*"):ti,ab,kw OR (electroacupuncture):ti,ab,kw OR (neuro modulation):ti,ab,kw OR (electro*acupuncture):ti,ab,kw OR (neuromodulation):ti,ab,kw

#14 (transcutaneous electr* stimulat*):ti,ab,kw OR ("interferential electr* stimulat*"):ti,ab,kw

#15 MeSH descriptor: [Fertilization in Vitro] explode all trees

#16 (Baby, Test-Tube):ti,ab,kw OR (Test Tube Babies):ti,ab,kw OR (Test-Tube Baby):ti,ab,kw OR (Injection, Intracytoplasmic Sperm):ti,ab,kw OR (Injections, Intracytoplasmic Sperm):ti,ab,kw (Word variations have been searched)

#17 (Intracytoplasmic Sperm Injection):ti,ab,kw OR (Sperm Injection, Intracytoplasmic):ti,ab,kw OR (Intracytoplasmic Sperm Injections):ti,ab,kw OR (ICSI):ti,ab,kw OR (Injections, Sperm, Intracytoplasmic):ti,ab,kw (Word variations have been searched)

#18 MeSH descriptor: [Sperm Injections, Intracytoplasmic] explode all trees

#19 (In Vitro Fertilization):ti,ab,kw OR (In Vitro Fertilizations):ti,ab,kw OR (Test-Tube Fertilization):ti,ab,kw OR (Fertilization, Test-Tube):ti,ab,kw OR (Fertilizations, Test-Tube):ti,ab,kw (Word variations have been searched)

#20 (Test Tube Fertilization):ti,ab,kw OR (Test-Tube Fertilizations):ti,ab,kw OR (Fertilizations in Vitro):ti,ab,kw (Word variations have been searched)

#21 #1 OR #2 OR #3 OR #15 OR #16 OR #17 OR #18 OR #19 OR #20

#22 #4 OR #5 OR #6 OR #7 OR #8 OR #9 OR #10 OR #11 OR #12 OR #13 OR #14

#23 MeSH descriptor: [Endometrium] explode all trees

#24 MeSH descriptor: [Uterus] explode all trees

#25 MeSH descriptor: [Uterine Artery] explode all trees

#26 ("endometria"):ti,ab,kw OR ("endometrial"):ti,ab,kw OR (endometrial receptivity):ti,ab,kw OR (endometria rceptivity):ti,ab,kw OR (Uteri):ti,ab,kw (Word variations have been searched)

#27 ("womb"):ti,ab,kw OR (Wombs):ti,ab,kw OR (Uterus Cornua):ti,ab,kw OR (Uterine Cornua):ti,ab,kw OR (Cornua, Uterine):ti,ab,kw (Word variations have been searched)

#28 (Uterine Fundus):ti,ab,kw OR (Fundus, Uterine):ti,ab,kw OR (Fundus Uteri):ti,ab,kw OR (Fundus Uterus):ti,ab,kw OR (Uteri, Fundus):ti,ab,kw (Word variations have been searched)

#29 (Arteries, Uterine):ti,ab,kw OR (Artery, Uterine):ti,ab,kw OR (Uterine Arteries):ti,ab,kw OR (endometrium blood flow):ti,ab,kw OR (thin endometrium):ti,ab,kw (Word variations have been searched)

#30 (endometrium thickness):ti,ab,kw OR (endometrium pattern):ti,ab,kw OR (endometrium contraction):ti,ab,kw OR (thin uterus):ti,ab,kw OR (pulse index):ti,ab,kw (Word variations have been searched)

#31 #21 AND #22

#32 (pulse index):ti,ab,kw OR (resistive index):ti,ab,kw OR ("uterine artery"):ti,ab,kw OR ("uterine blood flow"):ti,ab,kw

#33 (endometri*):ti,ab,kw OR (uter*):ti,ab,kw

#34 MeSH descriptor: [Clinical Trials as Topic] explode all trees

#35 (randomized controlled trial):pt OR (randomized controlled trial):ti,ab,kw OR (random allocation):ti,ab,kw (Word variations have been searched)

#36 #23 OR #24 OR #25 OR #26 OR #27 OR #28 OR #29 OR #30 OR #32 OR #33

#37 #34 OR #35

#38 #31 AND #36 AND #37

**Web of Science** **search strategy (Feb 24, 2022)= 55**

#1 TS=(infertility OR Sterility, Reproductive OR Sterility Reproductive OR Sterility OR Subfertility OR Sub-Fertility OR infertil* OR subfertil* OR ‘Fertilization in Vitro’ OR ‘In Vitro Fertilization’ OR ‘In Vitro fertilization’ OR Test-Tube OR Fertilization OR Fertilization, Test-Tube OR fertilization, Test-Tube OR Test Tube OR Fertilization Test-Tube OR fertilization OR fertilization in Vitro OR Test-Tube Babies OR Babies OR Test-Tube Baby OR Test-Tube OR Test Tube Babies OR Test-Tube Baby OR Sperm Injections, Intracytoplasmic OR Injection, Intracytoplasmic Sperm Injections OR Intracytoplasmic Sperm OR Intracytoplasmic Sperm Injection OR Sperm Injection, Intracytoplasmic OR Intracytoplasmic Sperm Injections OR ICSI OR Injections, Sperm, Intracytoplasmic)

#2 TS=(intracytoplasmic sperm injection* OR artificial insemination* OR assisted reproduct* OR ovulation induc* OR infertil* OR assisted reproduct* OR ovari* adh2 stimulat*)

#3 #1 OR #2

#4 TS=(Acupuncture OR Pharmacopuncture OR Acupuncture Therapy OR Acupuncture Treatment OR Acupuncture Treatments OR Treatment, Acupuncture OR Therapy, Acupuncture OR Pharmacoacupuncture Treatment OR Treatment, Pharmacoacupuncture OR Pharmacoacupuncture Therapy OR Therapy, Pharmacoacupuncture OR autotomy OR anulotomies OR Acupuncture Points OR Acupuncture Point OR Point, Acupuncture OR Points, Acupuncture OR Acupoints OR Acupoint OR Acupressure OR Shiatsu OR Zhi Ya OR chip Ya OR shiatsu)) OR TS=(meridian OR meridian* OR Ching Lo OR Jing Luo OR Luo, Jing OR Jingluo)) OR TS=(acup*?point* OR acup* OR trigger adj3 point*)

# 5 TS=(Transcutaneous Electric Nerve Stimulation OR Electric Stimulation, Transcutaneous OR transcutaneous electr* stimulat* OR nerve stimulat* OR electro‐acupuncture OR electroacupuncture OR neuro‐modulation OR neuromodulation OR trans‐abdominal stimulat* OR sacral nerve stimulat* OR interferential electr* stimulat* OR Electric Stimulation, Transcutaneous OR Electric Stimulation, Transcutaneous OR Percutaneous Electrical Nerve Stimulation OR Transcutaneous Electrical Nerve Stimulation OR Transcutaneous Nerve Stimulation OR Percutaneous Neuromodulation Therapy OR Neuromodulation Therapy, Percutaneous OR Neuromodulation*, Percutaneous Electrical)

#6 #4 OR #5

#7 (TS=(Endometrium OR endometrial OR endometrium receptivity OR endometrial receptivity OR uterus OR Uteri OR Womb OR Wombs OR Uterus Cornua OR Uterine Cornua OR Cornua, Uterine OR Uterine Fundus OR Fundus, Uterine OR Fundus Uteri OR Fundus Uterus OR Uteri, Fundus OR uterine arteries OR Arteries, Uterine OR Artery, Uterine OR Uterine Arteries OR endometrium blood flow OR pulse index OR resistive index )) OR TS=(endometri* OR uter*)

#8 (TS=(clinical trials as topic OR clinical trial OR random allocation OR RCT OR clin* OR randmization*) OR SO=(clin* OR randmization*))

#9 #3 OR #6 OR #7 OR #8

**EMBASE search strategy search strategy (Feb 24, 2022)=38**

#1 'infertility'/exp OR 'fertility disorder':ti,ab,kw OR 'infecundity':ti,ab,kw OR 'infertility':ti,ab,kw OR 'primary infertility':ti,ab,kw OR 'secondary infertility':ti,ab,kw OR 'sexual sterility':ti,ab,kw OR 'sterility, sexual':ti,ab,kw OR 'sterility, reproductive':ti,ab OR 'sterility'/exp OR 'reproductive sterility':ti,ab OR 'subfertility'/exp OR 'fertility, reduced':ti,ab,kw OR 'fertility, sub':ti,ab,kw OR 'hypofertility':ti,ab,kw OR 'reduced fertility':ti,ab,kw OR 'sub-fertility':ti,ab,kw OR 'subfertility':ti,ab,kw OR 'sub fertility':ti,ab OR 'in vitro fertilization'/exp OR 'test-tube fertilization':ti,ab OR 'fertilization, test-tube':ti,ab OR 'test tube fertilization':ti,ab OR 'test-tube babies':ti,ab OR 'babies, test-tube':ti,ab OR 'test tube babies':ti,ab OR 'sperm injections, intracytoplasmic':ti,ab OR 'intracytoplasmic sperm injection'/exp OR 'sperm injection, intracytoplasmic':ti,ab OR 'intracytoplasmic sperm injections':ti,ab OR icsi:ti,ab OR 'injections, sperm, intracytoplasmic':ti,ab OR 'intracytoplasmic sperm injection*':ti,ab,kw OR 'artificial insemination*':ti,ab,kw OR 'ovulation induc*':ti,ab,kw OR infertil*:ti,ab,kw OR 'assisted reproduct*':ti,ab,kw OR 'ovari* adh2 stimulat*':ti,ab,kw

#2. 'acupuncture'/exp OR 'acupuncture':ti,ab,kw OR 'acupuncture therapy':ti,ab,kw OR 'shonishin':ti,ab,kw OR 'acupuncture point'/exp OR 'point, acupuncture':ti,ab OR 'points, acupuncture':ti,ab OR acupoints:ti,ab OR acupoint:ti,ab OR 'pharmacopuncture'/exp OR 'herb acupuncture':ti,ab,kw OR 'herbal acupuncture':ti,ab,kw OR 'herbalized acupuncture':ti,ab,kw OR 'pharmaco-acupuncture':ti,ab,kw OR 'pharmaco-puncture':ti,ab,kw OR 'pharmacoacupuncture':ti,ab,kw OR 'pharmacopuncture':ti,ab,kw OR 'acupuncture therapy':ti,ab OR 'acupuncture treatment':ti,ab OR 'treatment, acupuncture':ti,ab OR 'pharmacoacupuncture treatment':ti,ab OR 'treatment, pharmacoacupuncture':ti,ab OR 'pharmacoacupuncture therapy':ti,ab OR 'therapy, pharmacoacupuncture':ti,ab OR 'acupotomy'/exp OR acupotomies:ti,ab OR 'acupressure'/exp OR 'acupressure':ti,ab,kw OR 'shiatsu'/exp OR 'shiatsu':ti,ab,kw OR 'zhi ya':ti,ab OR 'chih ya':ti,ab OR shiatzu:ti,ab OR 'meridian'/exp/mj OR meridian*:ti,ab,kw OR 'ching lo':ti,ab,kw OR 'jing luo':ti,ab,kw OR 'luo, jing':ti,ab,kw OR jingluo:ti,ab,kw OR acup*?point*:ti,ab,kw OR acup*:ti,ab,kw OR 'trigger adj3 point*':ti,ab,kw

#3 'transcutaneous electric nerve stimulation':ti,ab,kw OR 'transcutaneous electr* stimulat*':ti,ab,kw OR 'nerve stimulat*':ti,ab,kw OR electro‐acupuncture:ti,ab,kw OR electroacupuncture:ti,ab,kw OR neuro‐modulation:ti,ab,kw OR neuromodulation:ti,ab,kw OR 'trans‐abdominal stimulat*':ti,ab,kw OR 'sacral nerve stimulat*':ti,ab,kw OR 'interferential electr* stimulat*':ti,ab,kw OR 'electric stimulation, transcutaneous':ti,ab,kw OR 'percutaneous electrical nerve stimulation':ti,ab,kw OR 'transcutaneous electrical nerve stimulation':ti,ab,kw OR 'transcutaneous nerve stimulation':ti,ab,kw OR 'percutaneous neuromodulation therapy':ti,ab,kw OR 'neuromodulation therapy, percutaneous':ti,ab,kw OR 'neuromodulation*, percutaneous electrical':ti,ab,kw

#4 'endometrium'/exp OR 'endometrial epithelium':ti,ab,kw OR 'endometrium':ti,ab,kw OR 'endometrium epithelium':ti,ab,kw OR 'endometrium extract':ti,ab,kw OR 'tunica mucosa uteri':ti,ab,kw OR 'uterine endometrium':ti,ab,kw OR 'uterine mucosa':ti,ab,kw OR 'uterus endometrium':ti,ab,kw OR 'uterus mucosa':ti,ab,kw OR endometria:ti,ab OR 'endometria`l receptivity':ti,ab OR 'endometria rceptivity':ti,ab OR 'uterus cancer'/exp OR uteri:ti,ab OR 'wombat'/exp OR 'uterus cornua':ti,ab OR 'uterine fundus'/exp OR 'fundus, uterine':ti,ab OR 'fundus uteri':ti,ab OR 'fundus uterus':ti,ab OR 'uteri, fundus':ti,ab OR 'arteries, uterine':ti,ab OR 'uterine artery'/exp OR 'uterine arteries':ti,ab OR 'endometrium blood flow'/exp OR 'pulse index':ti,ab OR 'resistive index'/exp OR endometri*:ti,ab,kw OR uter*:ti,ab,kw

#5 'clinical trial (topic)'/exp OR 'randomization'/exp OR 'random allocation' OR 'randomisation' OR 'randomization' OR rct:ti,ab

#6. #2 OR #3

#7. #1 AND #4 AND #5 AND #6

**Clinicaltrail.gov strategy search strategy (Feb 25, 2022)=4**

(Acupuncture OR Pharmacopuncture OR Acupuncture Therapy OR Acupuncture Treatment OR Acupuncture Treatments OR Treatment, Acupuncture OR Therapy, Acupuncture OR Pharmacoacupuncture Treatment OR Treatment, Pharmacoacupuncture OR Pharmacoacupuncture Therapy OR Therapy, Pharmacoacupuncture OR autotomy OR anulotomies OR Acupuncture Points OR Acupuncture Point OR Point, Acupuncture OR Points, Acupuncture OR Acupoints OR Acupoint OR Acupressure OR Shiatsu OR Zhi Ya OR chip Ya OR shiatsu OR meridian OR meridian* OR Ching Lo OR Jing Luo OR Luo, Jing OR Jingluo OR acup*?point* OR acup* OR trigger adj3 point* OR Transcutaneous Electric Nerve Stimulation OR Electric Stimulation, Transcutaneous OR transcutaneous electr* stimulat* OR nerve stimulat* OR electro‐acupuncture OR electroacupuncture OR neuro‐modulation OR neuromodulation OR trans‐abdominal stimulat* OR sacral nerve stimulat* OR interferential electr* stimulat* OR Electric Stimulation, Transcutaneous OR Electric Stimulation, Transcutaneous OR Percutaneous Electrical Nerve Stimulation OR Transcutaneous Electrical Nerve Stimulation OR Transcutaneous Nerve Stimulation OR Percutaneous Neuromodulation Therapy OR Neuromodulation Therapy, Percutaneous OR Neuromodulation*, Percutaneous Electrical) AND (Endometrium OR endometrial OR endometrium receptivity OR endometrial receptivity OR uterus OR Uteri OR Womb OR Wombs OR Uterus Cornua OR Uterine Cornua OR Cornua, Uterine OR Uterine Fundus OR Fundus, Uterine OR Fundus Uteri OR Fundus Uterus OR Uteri, Fundus OR uterine arteries OR Arteries, Uterine OR Artery, Uterine OR Uterine Arteries OR endometrium blood flow OR pulse index OR resistive index OR endometri* OR uter*) | Interventional Studies | infertility OR Sterility, Reproductive OR Sterility Reproductive OR Sterility OR Subfertility OR Sub-Fertility OR infertil* OR subfertil* OR ‘Fertilization in Vitro’ OR ‘In Vitro Fertilization’ OR ‘In Vitro fertilization’ OR Test-Tube OR Fertilization OR Fertilization, Test-Tube OR fertilization, Test-Tube OR Test Tube OR Fertilization Test-Tube OR fertilization OR fertilization in Vitro OR Test-Tube Babies OR Babies OR Test-Tube Baby OR Test-Tube OR Test Tube Babies OR Test-Tube Baby OR Sperm Injections, Intracytoplasmic OR Injection, Intracytoplasmic Sperm Injections OR Intracytoplasmic Sperm OR Intracytoplasmic Sperm Injection OR Sperm Injection, Intracytoplasmic OR Intracytoplasmic Sperm Injections OR ICSI OR Injections, Sperm, Intracytoplasmic OR intracytoplasmic sperm injection* OR artificial insemination* OR assisted reproduct* OR ovulation induc* OR infertil* OR assisted reproduct* OR ovari* adh2 stimulat*

**CNKI search strategy (Feb 25, 2022) =108**

(SU=体外受精+试管婴儿+辅助生殖+胚胎移植+不孕+不孕症 +IVF+ICSI+胞浆内精子注射) AND (SU=针灸+针灸疗法+针法+针刺+灸法+经皮神经电刺激+穴位疗法 +穴位+电针+耳穴+穴位刺激+经络+腧穴) AND (TKA=子宫内膜厚度+子宫内膜+子宫容受+子宫内膜容受+子宫动脉+子宫动脉血流+子宫内膜血流+子宫内膜形态+子宫内膜收缩+子宫+动脉阻力+动脉搏动+内膜指数) AND(TKA=随机对照试验+临床研究+临床试验+RCT)

**Wanfang Database search strategy (Feb 25, 2022) =164**

全部:(体外受精 OR 试管婴儿 OR 辅助生殖 OR 胚胎移植 OR 不孕 OR 不孕症 OR IVF OR ICSI OR 胞浆内精子注射 OR 精子注射 ) and 全部:(针灸 OR 针灸疗法 OR 针法 OR 针刺 OR 灸法 OR 经皮神经电刺激 OR 穴位疗法 OR 穴位 OR 电针 OR 耳穴 OR 穴位刺激 OR 经络 OR 腧穴) and 全部:(子宫内膜厚度 OR 子宫内膜 OR 子宫容受 OR 子宫内膜容受 OR 子宫动脉 OR 子宫动脉血流 OR 子宫内膜血流 OR 子宫内膜形态 OR 子宫内膜收缩 OR 子宫 OR 动脉阻力 OR 动脉搏动 OR 内膜指数) and 全部:(随机对照试验 OR 临床研究 OR 临床试验 OR RCT)

**CBM search strategy (Feb 25, 2022) =160**

("体外受精"[常用字段:智能] OR "试管婴儿OR 辅助生殖"[常用字段:智能] OR "胚胎移植OR 不孕"[常用字段:智能] OR "不孕症"[常用字段:智能] OR "IVF"[常用字段:智能] OR "ICSI"[常用字段:智能] OR "胞浆内精子注射"[常用字段:智能]) AND( "针灸疗法"[常用字段:智能] OR "针刺"[常用字段:智能] OR "针刺"[常用字段:智能] OR "灸法"[常用字段:智能] OR "经皮神经电刺激"[常用字段:智能] OR "穴位疗法"[常用字段:智能] OR "穴位"[常用字段:智能] OR "针灸"[常用字段:智能] OR "电针"[常用字段:智能] OR "耳穴"[常用字段:智能] OR "穴位刺激"[常用字段:智能]) AND( "子宫内膜厚度"[常用字段:智能] OR "子宫内膜"[常用字段:智能] OR "子宫容受"[常用字段:智能] OR "子宫内膜容受"[常用字段:智能] OR "子宫动脉"[常用字段:智能] OR "子宫内膜血流"[常用字段:智能] OR "子宫内膜形态"[常用字段:智能] OR "子宫内膜收缩"[常用字段:智能] OR "动脉阻力" OR[常用字段:智能] "动脉搏动" [常用字段:智能] OR "内膜指数" [常用字段:智能]) AND( "随机对照试验"[常用字段:智能] OR "临床研究"[常用字段:智能] OR "临床试验"[常用字段:智能] OR "RCT"[常用字段:智能])

**VIP search strategy (Feb 25, 2022) =****12**

(题名或关键词:(体外受精 OR 试管婴儿OR 辅助生殖 OR 胚胎移植OR 不孕 OR 不孕症 OR IVF OR ICSI OR 胞浆内精子注射))AND (题名或关键词:(针灸疗法OR 针刺OR灸法OR经皮神经电刺激OR穴位疗法OR穴位OR针灸OR电针OR耳穴OR穴位刺激)) AND (题名或关键词:(子宫内膜厚度 OR 子宫内膜 OR 子宫容受 OR 子宫内膜容受 OR 子宫动脉 OR 子宫动脉血流 OR 子宫内膜血流 OR 子宫内膜形态 OR 子宫内膜收缩 OR 子宫 OR 动脉阻力 OR 动脉搏动 OR 内膜指数)) AND (题名或关键词: (随机对照试验 OR 临床研究 OR 临床试验 OR RCT))
