# Supplementary figures and images for: The Dose-Related Efficacy of Acupuncture on Endometrial Receptivity in Infertile Women: A Systematic Review and Meta-Analysis
Source: Front Public Health. 2022 Apr 28;10:858587. doi: 10.3389/fpubh.2022.858587 (PMC9095926; doi:10.3389/fpubh.2022.858587)

## Slide 1
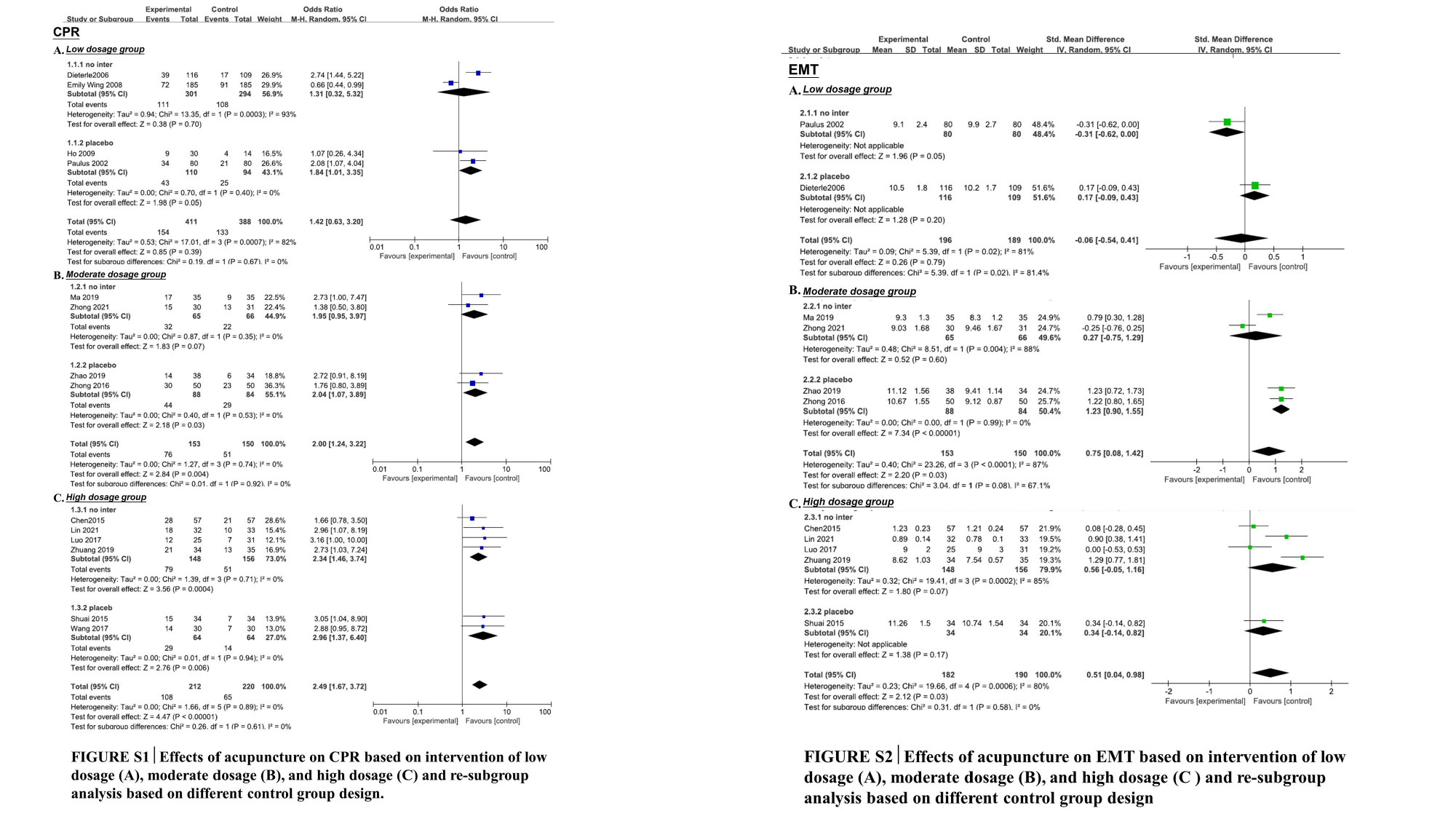

## Slide 2
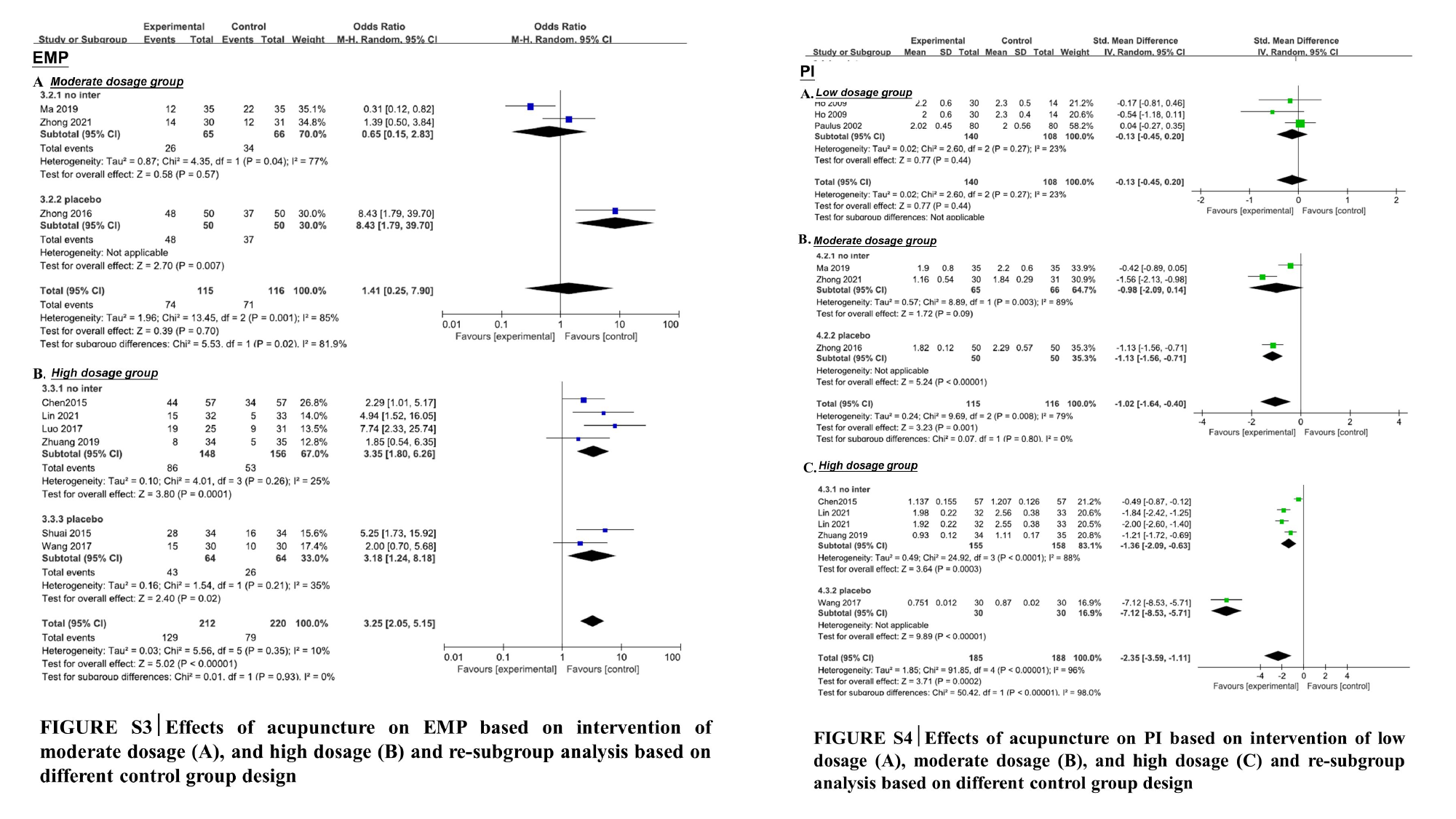

## Slide 3
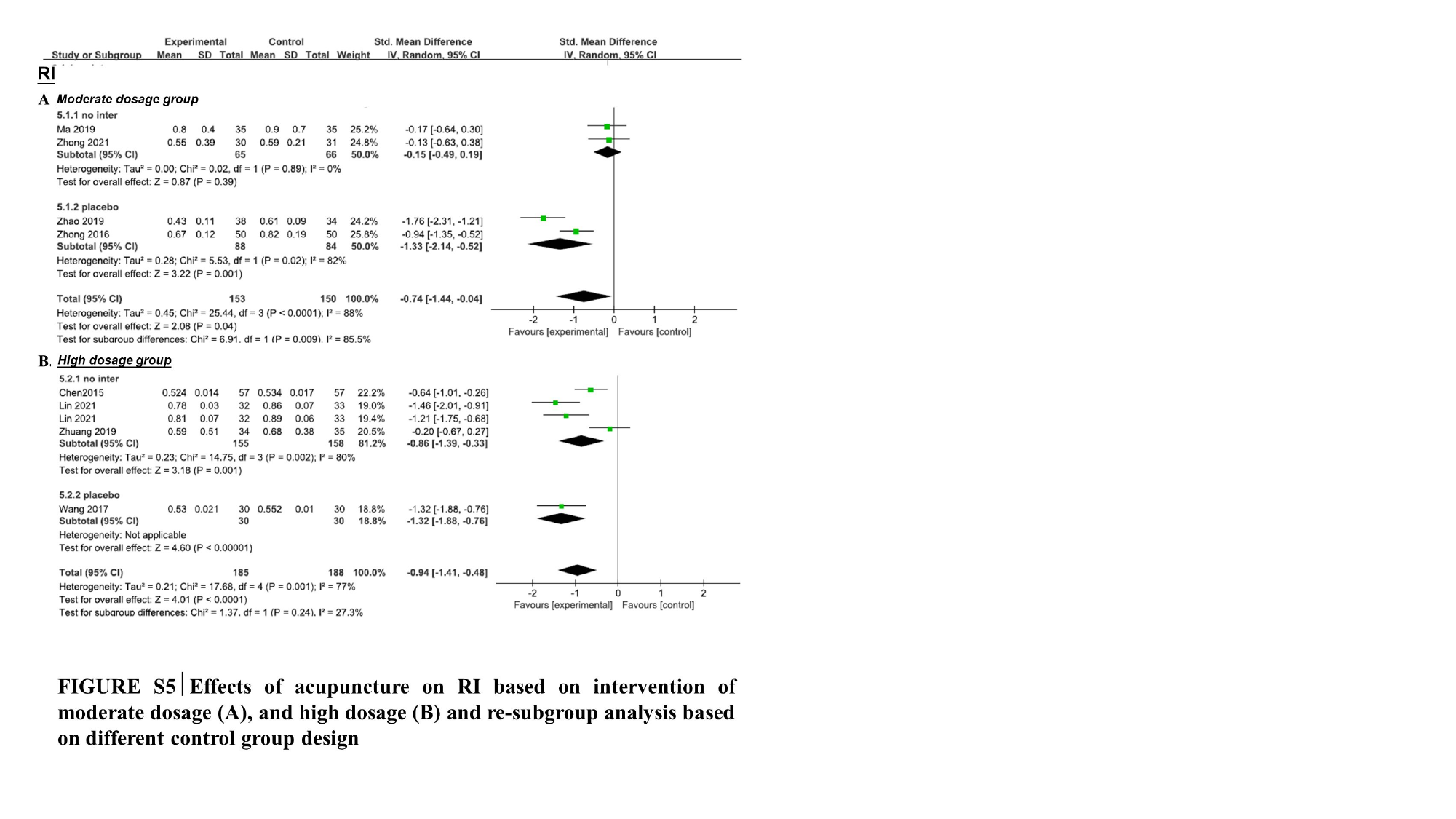

## Slide 4
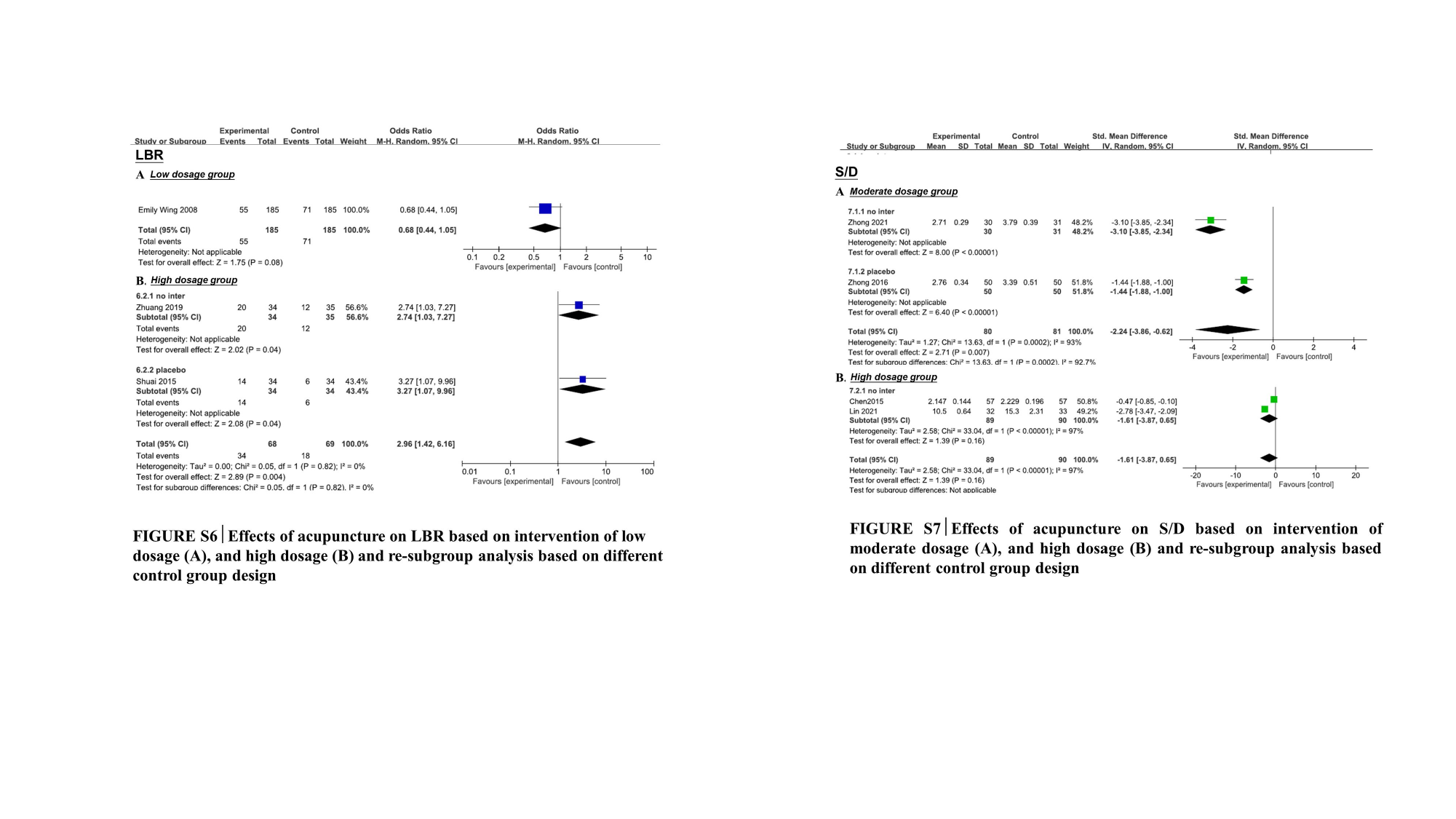

Supplement: Supplementary file 3 [file Presentation_1.PPTX]
